# Environmental structure drives resistance to phages and antibiotics during phage therapy and to invading lysogens during colonisation

**DOI:** 10.1038/s41598-019-39773-3

**Published:** 2019-02-28

**Authors:** Jorge A. Moura de Sousa, Eduardo P. C. Rocha

**Affiliations:** Microbial Evolutionary Genomics, Institut Pasteur, CNRS, UMR3525, Paris, 75015 France

## Abstract

Microbial communities are shaped by bacteriophages through predation and lysogeny. A better understanding of the interactions between these processes across different types of environments is key to elucidate how phages mediate microbial competition and to design efficient phage therapies. We introduce an individual-based model (eVIVALDI) to investigate the role of environmental structure in the elimination of a population with a combined treatment of antibiotics and virulent phages, and in the invasion of a population of phage-sensitive bacteria by lysogens. We show that structured environments facilitate the emergence of double resistance, to antibiotics and phages, due to limited diffusion of phage particles and increased nutrient availability from dead cells. They also hinder phage amplification, thus decreasing the generation of phage genetic diversity and increasing the unpredictability of phage-bacteria arms-races. We used a machine learning approach to determine the variables most important for the invasion of sensitive populations by lysogens. They revealed that phage-associated traits and environmental structure are the key drivers of the process. Structured environments hinder invasions, and accounting for their existence improves the fit of the model to published *in vivo* experimental data. Our results underline environmental structure as key to understand *in vivo* phage-bacteria interactions.

## Introduction

Microbial organisms are pervasive across all natural environments, including the human body. Their adaptation and organization in communities may lead to disease^1^, drive host evolution^2^, and produce major changes in ecosystems^3,4^. Ecological interactions in microbial communities influence, and are influenced by, the rapid pace with which microbes acquire adaptive changes^5,6^. A striking example is the relationship between bacteria and bacteriophages (from here on referred to as phages), because the latter predate on the former whilst also driving their adaptation^4^. Phages are the most abundant entities in nature^7,8^ and very efficient bacterial predators; it has been estimated that they promote the turnover of ~20% of bacterial mass every single day in certain environments^9,10^. In the context of widespread antibiotic resistance, this has led to a rekindled interest in phage therapy as an adjuvant or a replacement of antibiotic therapy against multi-resistant bacteria^11^.

Virulent phages follow a strictly lytic cycle within their hosts, whereas temperate phages opt between the lytic cycle and lysogeny. Lysogens are resistant to novel infections by the phage (superinfection exclusion), and sometimes other phages, impeding the use of temperate phages to extinguish bacterial populations^12–14^. Bacterial pathogens are more likely to be lysogens^15^, and many of their temperate phages encode virulence factors^16^. Even if this has restricted the development of phage therapy to the use of virulent phages, the role of temperate phages in this process is important because virions arising from prophage induction can infect closely related competitor bacteria that are non-lysogenic for the phage, decreasing bacterial competition, increasing prophage frequency, and liberating resources for the growth of the remaining lysogens^17^. As a result, prophage induction facilitates the colonization of communities of sensitive bacteria by invading lysogens^13,18^.

Many experimental approaches have clarified the mechanisms underlying phage-bacteria interactions, usually on simplified^8,19^ but sometimes also on complex^20^ environments. *In vivo* studies of these interactions (*e.g*., in mammalian hosts^21^) tackle more natural environments, but have limited resolution in tracking temporal dynamics or the effects of individual mechanisms. Mathematical modelling provides a complementary approach to the study of phage-bacteria interactions, providing important insights on their co-evolutionary processes^22^ or the dynamics of particular bacterial defense mechanisms^23,24^. Previous studies have used deterministic models focused on individual mechanisms of interaction in simple environments, *e.g*., how the evolution of resistance to phage can affect clinical treatments^25^. These approaches have provided important insights on phage-bacteria interactions and can often be confronted with *in vitro* experiments. They also have the advantages of providing analytical solutions and using well-established techniques to explore the space of parameters. Yet, these models become much more complex, or even intractable, when tackling multiple mechanisms, spatial heterogeneity, and intrinsically stochastic process (such as mutation-selection-drift and lysis-lysogeny decisions), as found in most natural communities^26–30^. This may explain why these models sometimes fail to fully reproduce *in vivo* dynamics of phage infection^19^, or the lack of theoretical studies regarding the effects of treatments using both virulent phages and antibiotic to curb bacterial infections in more realistic (structured) environments^19,31,32^. Spatial structure affects the ability of individuals and antibiotics to diffuse freely in the environment^27,28^, and is known to shape the co-evolution of species involved in host-parasite relationships^33^. Not accounting for it has been regarded as a main limitation of previous models of phage-bacteria interactions^19,31^.

Individual (or agent) based models (IBMs) provide an interesting alternative to classical modelling approaches, by incorporating different (and potentially interacting) mechanisms at the level of the individual. This has made them successful standard modelling approaches in Ecology^34^, Social Sciences^35^, and now Microbiology, where they have been particularly useful to study complex microbial systems^36^. IBMs have been used to understand the effect of spatial structure in microbial social evolution^37^, the evolutionary dynamics of CRISPR-Cas systems^38^, and the role of spatial structure on the selection for antibiotic resistant bacteria^39^. Recently, IBM’s have also been used to better understand the role of structured environments in the interactions between virulent phages and bacteria^28,40–42^. Since population-level dynamics in IBMs emerge naturally from the collective individual behaviors, they are particularly appealing to investigate phage-bacteria interactions. At the individual level, mechanistic and stochastic decisions (e.g., the lysis-lysogeny decision of temperate phages) can be represented with biological detail, while the population level dynamics resulting from predation and lysogeny emerge from the interactions between the individual entities. Importantly, these models are inherently spatial, and provide a framework through which the role of environmental structure can be easily assessed.

We developed an IBM approach to study the multiple roles of phages in microbial communities under different types of environments: eVIVALDI – eco-**eV**olutionary m**I**crobial indi**V**idu**AL**-base**D** s**I**mulations. The model includes a genetic component, thus allowing to couple ecological and evolutionary dynamics, which has been less thoroughly explored in the context of structured environments^33^. We focus on two questions that are relevant for bacterial evolution and phage therapy, and compare their dynamics between well-mixed or structured environments. First, we study the ecological interactions between virulent phages and bacteria under phage and antibiotic pressure in either well-mixed or structured environments. This approach has been the object of many experimental studies, and pioneering modelling^31^, but its expected dynamics have not been investigated in structured environments. Second, we introduce lysogeny and super-infection exclusion in the model to study the role of prophage induction in bacterial competition. While this has been investigated before, both theoretically and experimentally^17,18,43,44^, most models have not accounted for population or environmental structure and have shown discrepancies with the results *in vivo*^19,21^. We show that considering structured environments influences the outcome of combined treatments with antibiotics and phage in multi-species communities, and enhances the acquisition of resistance mutations. We find also that structured environments, when compared to well-mixed environments, lead to a better fit of previous experimental results of the effects of lysogeny *in vivo*.

## Methods

### Concept and basic implementation

The eVIVALDI model was developed in Python (version 2.7.3), using an object-oriented approach, with a focus on the flexibility and extensibility of mechanisms and parameters simulated. Here, we describe the main processes and parameters of the model. The complete ODD (Overview, Design concepts, and Details) protocol^45^ of the model is available as supplementary text (Text S1). It includes the details of the simulations and the list and explanation of all the parameters modelled in this manuscript and their possible values (Table S1). The source of the software can be obtained in the following link: https://gitlab.pasteur.fr/jsousa/eVIVALDI. The simulations can be run on a typical desktop computer. In a 3 GHz 8-core Mac Pro, with 32GB of RAM, a replicate of a simulation (100 iterations), takes from ~5 to 30 minutes for a bacterial population of size up to 10^4^ and a phage population of size of up to 10^6^, depending also on the other parameters. Computations can also be performed in a cluster, allowing the parallel simulation of multiple parameters.

### Entities and their ecological setup

**Figure 1:**
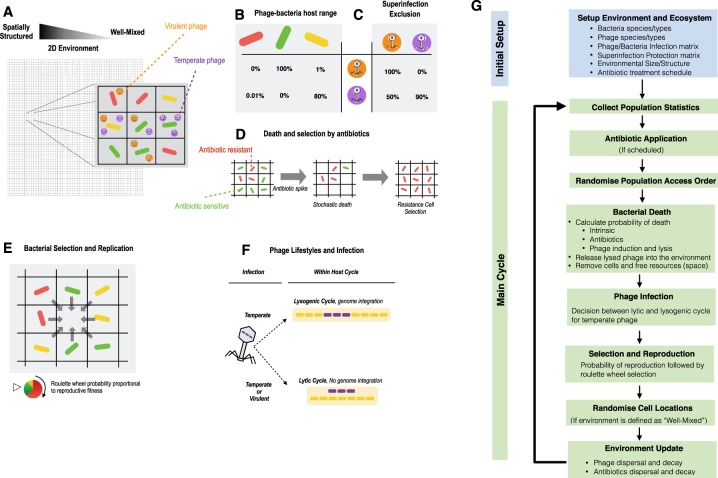
Mechanisms and workflow of the eVIVALDI model. (**A**) Bacterial cells and bacteriophage particles are modelled in a 2-dimensional space, where each (x,y) location holds at most a single bacterial cell and at most a predefined maximum number of phages. The environment ranges from completely well-mixed (liquid), where the contents of each location are randomized at each iteration, to spatially structured, where they are fixed. An intermediate structure is achieved by allowing replication of bacterial cells into a neighbourhood of a given distance and by the diffusion of viral particles within a variable radius (corresponding to the amount of structure simulated). Bacteria and phage can be of different species, and the latter exist as entities either in the environment, where they can infect new hosts, or within hosts, where they either replicate or integrate into their genomes. (**B**) Phage host range is defined in a matrix where each phage has a probability of infecting a given bacterial species, corresponding also to the adorption probability of the phage relative to that host. (**C**) Superinfection exclusion is the probability that infection by a given phage aborts when a given type of prophage is present. (**D**) The basal probability of bacterial death can increase by antibiotic exposure or phage infection. Phages decay in function of the period of time spent outside a bacterial host. (**E**) Bacteria compete to reproduce to empty locations, with the fittest bacteria being more likely to produce an offspring. The offspring inherits the traits of the parent cell, but can undergo mutations and is placed into the free location. (**F**) The type of phage infection is determined by the lifestyle of the phage, with virulent phage following an obligatory lytic cycle, whilst temperate phage can undertake the lytic or the lysogenic cycle following a stochastic decision affected by the density of phages in the environment. (**G**) The main cycle of a typical simulation within the model. See complete ODD in Supplementary Material (Text S1).

Both bacterial cells and phage particles are represented as independent individuals on an environment represented as a two-dimensional grid with Moore neighbourhood (the 8 connected grid spaces of each location, for a Moore distance of one) (Fig. 1A). Bacteria can be of different species. Each individual bacterium has a genome with essential, accessory and, eventually, prophage genes. Bacteria have individual phenotypes, such as growth rate or the ability to survive antibiotic exposure. Phages can be from different species, have different lifestyles (temperate, virulent or defective), and possess individual phenotypes (e.g., attachment receptors and burst sizes). The host range of phage hosts is defined by a matrix (Fig. 1B), and the superinfection exclusion rules amongst phages is defined in a similar way (Fig. 1C).

### Environmental and bacterial updates

The environment and the individuals are updated and behave according to biologically inspired rules. The environment can be completely structured, semi-structured or not structured at all (i.e., well-mixed), and it can be set as bounded or have a toroidal space. The type of structure influences the diffusion of the different bacterial cells and environmental particles (phage and antibiotics). Each location can hold a single bacterial cell and several phage cells. Simulation time proceeds with time-steps defined as iterations. The classical notion of generation at the population level is not explicitly parameterized, but is instead a consequence of birth-death processes at the level of each individual cell (see Text S1, section 3.3.7). Thus, the time equivalent of an iteration, and its equivalence to a “bacterial generation”, is dependent on the parameters used to characterize the bacteria. Free space is the bacterial resource to be consumed, and it is freed whenever bacteria die. Bacterial death can be intrinsic (e.g., of old age) or explicit (e.g., exposure to antibiotics or predation by phage) (Fig. 1D). When a free space is available, the neighboring bacteria compete for reproduction. The outcome of the competition is chosen through a roulette wheel method that accounts for the fitness of each bacterium. The successful bacterium generates an offspring into the free space (Fig. 1E). Reproduction is therefore asynchronous across the population. Bacteria can be infected by phage in the environment. The outcome of the infection depends on the phage lifestyle and, for temperate phage, the lysis-lysogeny decision (Fig. 1F). This decision is stochastic but influenced by the number of surrounding phages. For temperate phage, integration in the host genome means vertical inheritance with host replication, until the phage excises from the genome, according to a probability that can be low but non-null throughout the simulation (stochastic prophage induction) and that can also be influenced by the level of antibiotic stress to which the host is exposed.

### Input, output and documentation of the model

The inputs of each simulation are two text files that define the general parameters and also the ecological setup of the environment (types and numbers of bacteria and/or phage, along with their attributes). The statistics collected at different time points are stored in dictionaries and dataframes (using the library *pandas*), can be tailored to the experimenter’s choice and can be represented visually (using the libraries *matplotlib* and *seaborn*) or outputted to a file.

### Random Forest Analysis

The Random Forest Analysis (RFA) is based on simulations performed with the model, covering 3000 random combinations of parameters, with 30 simulated repeats per combination. The output of this cohort of simulations is grouped and resumed in response variables. This results in a large table with input parameters and response variables to which we add a column with 3000 rows of a random parameter (i.e., a choice of a number between 1 and 3). This table is used as input of the randomForest package in R (version 4.6.12), where the *randomForest* function is run with the parameters ntrees set to 10000. The relative importance of each parameter (the percentage increase in minimum squared error, %IncMSE) is assessed using the *importance* function from the same package. This function evaluates the effect of excluding each parameter on the ability of the RFA to predict the response variable (the number of lysogens among resident bacteria, in our study). In the context of RFA, exclusion is performed by randomly assigning values to a parameter, rather than those used for the actual simulations. The predictive ability is compared between the original data and the permuted data (with the “excluded” parameter) and parameters that lead to high increases in prediction error (increased MSE) are deemed of high importance. The purposefully random parameter included in our analysis allows to assess the impact of random noise in the quantification of the relative importance of the parameters. Non-important parameters have a small or null increase in MSE, similar to that of random noise.

## Results and Discussion

### Outcomes of simultaneous antibiotic and phage therapy depend on environmental structure

Sensitive bacteria are killed by antibiotics or predated by virulent phages, raising the frequency of resistant bacteria. These processes have been independently analyzed assuming well-mixed environments, and we started by verifying that eVIVALDI could reproduce key previous results of the effect of these stressors in separate^31^ (for details, see Fig. S1). Yet, there is a lack of theoretical studies on the effects of combined treatments with antibiotics and phages in diverse bacterial communities, and particularly in structured environments. It was previously suggested that such treatments can increase the efficiency of antimicrobial therapy and decreased the emergence of resistances^46,47^. Here, we defined a simple community with one bacterial species sensitive to an antibiotic and resistant to a virulent phage, and another resistant to the antibiotic and sensitive to the virulent phage. Resistance (to antibiotics or phages) is costly and fixed (not allowed to evolve at this stage)^48^ (Fig. S2). Since both species have either of these traits, their relative fitness is similar, but see Fig. S3 for cases with dissimilar costs for each type of resistance.

**Figure 2:**
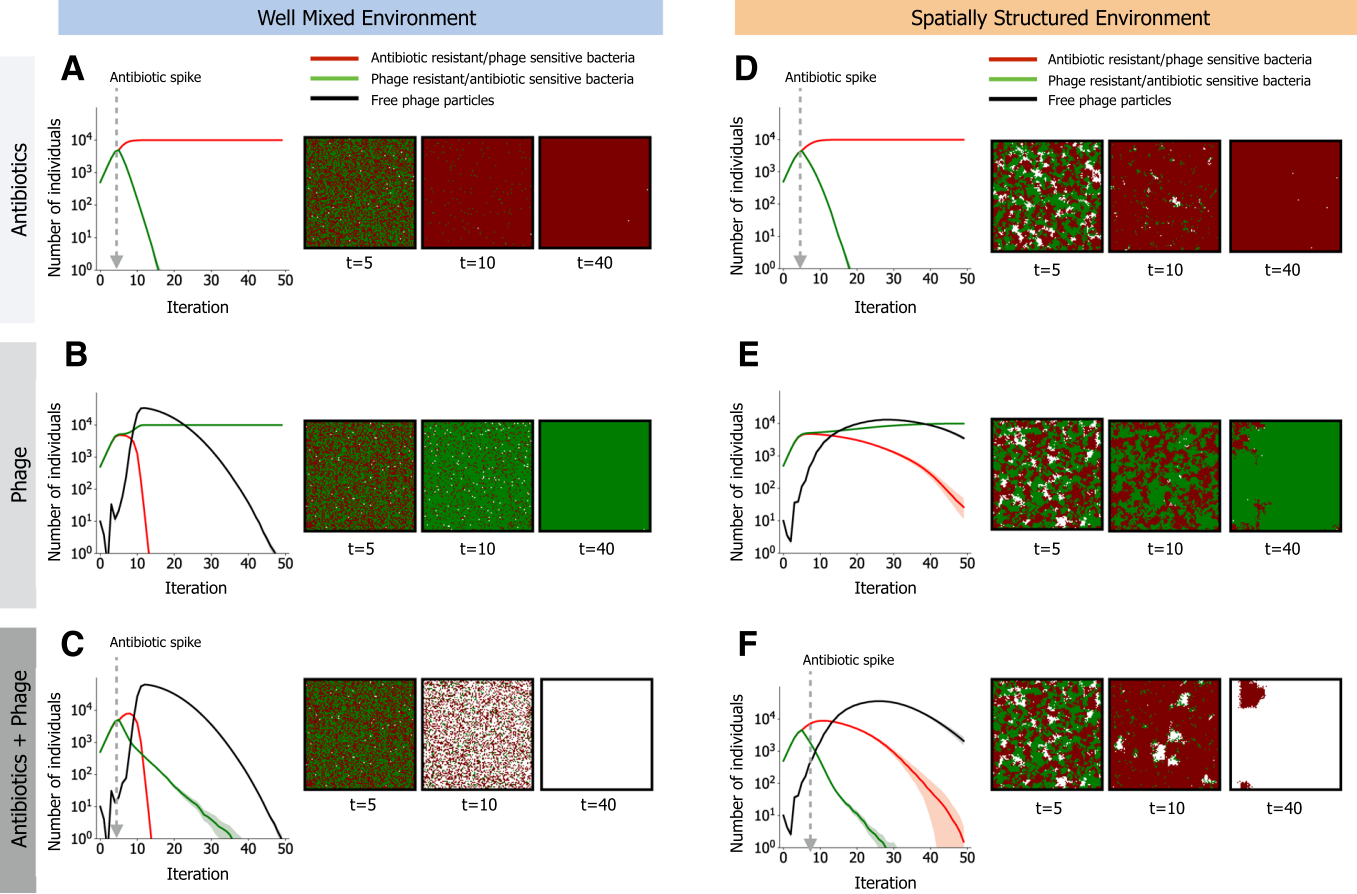
Community dynamics driven by antibiotic selection and phage predation. A small community composed of two different species is subjected to different selective pressures. Bacteria can be sensitive to antibiotics but resistant to phage (in green), or resistant to antibiotics but sensitive to phage (in red). We follow the temporal dynamics and show the populations in their respective colors (the number of free phage in the environment is shown in black). Solid lines indicate mean values for 30 simulations ran with the same parameters and shaded areas show their 95% confidence interval. At the right of each plot is a representative time lapse at 3 time points of the lattices for each scenario, where the colors represent each bacterial species and white spaces represent the absence of bacterial cells. In (**A**) and (**D**) antibiotics are applied at the indicated time. In (**B**) and (**E**), virulent phages (10 individual particles) are co-inoculated with the bacteria at time 0. In (**C**) and (**F**), both selective regimes are applied, with antibiotics applied at the indicated time and virulent phage co-inoculated with bacteria at time 0. In (**A**–**C**), the environment is homogeneous (well-mixed), as in liquid culture. In (**D**–**F**), the environment is spatially structured. In D) and F) antibiotics are applied homogenously in the structured environment, and in (**E** and **F**) each of the 10 phage particles is initially placed randomly in the biofilm. The complete set of parameters for these simulations is show in Supplementary Data 1.

We started by analyzing the result of the introduction of a slow-degrading antibiotic or a virulent phage in the environment (but not both). When antibiotics are introduced in well-mixed environments, antibiotic resistant bacteria rapidly increase to fixation (Fig. 2A). Changing the rates of antibiotic degradation only affects the kinetics of the process, not the overall qualitative behavior (Fig. S4). If antibiotics degrade fast, this usually implies the use of multiple doses in succession, which also does not fundamentally affect the dynamics of the process (Fig. S4M–P). For simplicity, we will focus on the case of a single slowly-degrading antibiotic dose in the rest of this study. When virulent phages are introduced instead of antibiotics (Fig. 2B), we observed an initial increase in the number of phages caused by the abundance of sensitive bacteria, but phage predation rapidly extinguished sensitive hosts leading to eventual extinction of phages themselves (black line in Fig. 2B).

Phage predation depends on the adsorption rate of viral particles to bacteria. In the model, this is defined as an individual probability for each phage particle. This can be converted into a population-level measure of adsorption rate - the one typically measured experimentally – by analyzing the population dynamics of the simulations (Fig. S5). We varied the individual adsorption rate to produce a range of frequencies of free phage particles between almost 0 and close to 100%. This allowed to define the relation between individual and population-level adsorption rates for this range of values. Expectedly, lower rates of adsorption result in delayed extinction of phage sensitive populations (Fig. S6). Extremely low values even resulted in phage extinction (Fig. S6E–H), but such low rates are unlikely to occur in nature when phages are well adapted to their host.

A combined treatment of antibiotics and virulent phages leads to the expected extinction of both bacterial populations, because none has, or can evolve, the ability to survive both selective pressures (Fig. 2C). However, the extinction of the antibiotic sensitive population is slower in the presence of phages (Fig. S7A), and can be prevented within the time-frame of our simulations when antibiotics have high degradation rates, even if they are administered periodically (Fig. S4M–P). This occurs due to the absence of competition for resources from antibiotic resistant cells, which are killed by the phage.

We then considered the effect of spatial structure on combinations of antibiotics and phage therapy. We assume that in structured environments there is no mixing or diffusion: bacteria are fixed until their death and divide to adjacent locations (Fig. 1E), whereas phages propagate by infecting nearby bacteria (see Text S1). When only antibiotics (and no phages) are applied homogeneously in the environment (i.e., all locations receive a similar concentration of antibiotics), the extinction of antibiotic sensitive bacteria is merely delayed in this environment, when compared to non-structured environments (Fig. 2D and Fig. S7B).

Heterogeneous distribution of antibiotics can occur in structured environments if antibiotics do not permeate all regions equally^49,50^. This is simulated in eVIVALDI by initially deploying antibiotics in a few locations (chosen randomly for each replicate simulation), which subsequently diffuse towards neighboring locations (see Text S1). This creates regions in the environment with low concentrations of antibiotics, and others where this concentration is very high. Because the former can be used as spatial refuges by bacteria sensitive to antibiotics, we observed long-term coexistence between sensitive and resistant bacteria (Fig. S8A), but only in the absence of phages (Fig. S8B).

Phage predation is also affected by the structure of the environment, because slow dispersion leads to “predation waves” producing spatial arrangements of dead cells akin to those observed in phage plaque assays (see Fig. S9 and Video S1). Ultimately, spatial structure results in delayed extinction of phage susceptible cells (Fig. 2E vs Fig. 2B). Interestingly, the presence of both phages and antibiotics leads to faster extinction of antibiotic sensitive populations in spatially structured than in well-mixed environments (Fig. 2F vs Fig. 2C, S7C), due to less efficient phage predation of their competitors. Hence, environmental structure can lead to inversions in the order of extinctions of both populations, by delaying the extinction of competitor strains. This suggests that even when the emergence of new resistance mutations is ignored (due to their cost or extreme rarity), ecological interactions can complicate the extrapolation of results obtained in well-mixed environments to *in vivo* situations.

### Emergence of bacteria resistant to antibiotics and virulent phage is enhanced in structured environments

**Figure 3:**
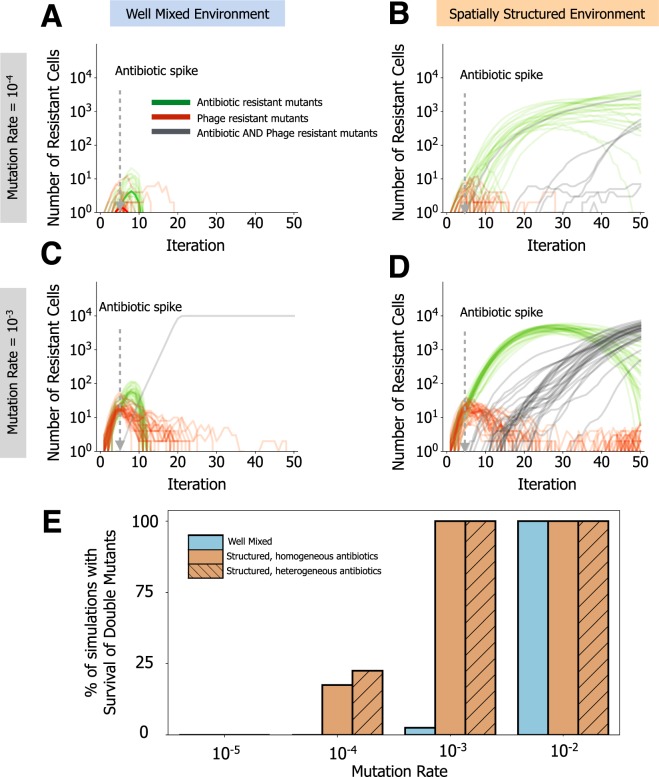
Spatial structure promotes the emergence of multi-resistant bacteria. (**A**–**D**) Simulations of a single bacterial species, initially sensitive to antibiotics and phage, evolving in the presence of both stressors. Lines show 30 replicate simulations with emerging resistant lineages (to one or both selective pressures). Single mutants resistant to phage are shown in red, whilst single mutants resistant to bacteria are shown in green. Double mutant lineages resistant to antibiotics and phage are shown in grey. Antibiotics are applied at the time point indicated by the arrow, whilst phages (10 particles) are inoculated at iteration 0 along with bacteria. In (**A** and **B**) resistant mutants emerge at a rate of 10^−4^ per cell per iteration (**C** and **D**) resistant mutants emerge at a rate of 10^−3^ per cell per iteration. (**A** and **C**) show dynamics from well-mixed environments. (**B** and **D**) show dynamics from spatially structured environments. (**E**) Percentage of simulations (out of 30) where lineages resistant both to antibiotics and phage have emerged, in either well mixed or spatially structured environments (with homogeneous or heterogeneous antibiotic exposure), for all the mutation rates tested (x-axis). The complete set of parameters for these simulations is show in Supplementary Data 1.

When we make our model more complex by allowing mutations towards resistance in initially sensitive bacteria, we observed stabilization of the bacterial populations, even if some of them still go extinct before or even after acquiring the adaptive mutation (due to the stochastic processes of mutation-selection-drift) (Fig. S10A,B). Under pressure of antibiotics and phages in well-mixed environments, double resistant cells emerge only when the mutation rate is very high (Fig. 3A and C, Fig. S10C). This is in agreement with experimental results identifying double mutants only in mutator strains^51^, and with observations that the presence of phages also reduces the rate of acquisition of antibiotic resistance^32^.

In structured environments, single resistant mutants, under a single stressor, increase at a slower frequency (Fig. 3B and D, Fig. S10D). In the presence of both stressors, single and especially double mutants (resistant to antibiotics and phage) are much more likely to emerge in structured environments, particularly when antibiotics are applied heterogeneously in the environment (Fig. 3E). This is caused by two effects. Firstly, the rare mutants resistant to antibiotics benefit from the resources available due to the death of the majority of bacterial population that is sensitive to these drugs. Secondly, phage particles amplify less efficiently in structured environments because their offspring are released locally and there are few hosts because of antibiotics. This leads to phage particles being stranded in the environment. The combination of these factors allows further replication of sensitive bacteria until they meet the stranded phage particles. This increases the span of time available for the acquisition of secondary mutations conferring resistance to phages, especially if the initial number of phages is not very high (Fig. S11).

The effect is enhanced when antibiotics are heterogeneously distributed in the environment because the number of surviving cells is higher. Although this increases their chance of contact with phages, spontaneous mutants can be generated at sufficiently higher frequency. Importantly, slower phage predation due to spatial structure is not sufficient to explain these observations, since in the absence of antibiotics, structured environments generate phage resistant mutants at a slower rate, compared to well-mixed environments (Fig. S10D vs Fig. S10C). Hence, the acquisition of multiple adaptive mutations conferring resistance to antibiotics and phages is more likely to occur in structured environments when there are sufficient resources for population expansion, and when antibiotic penetration is not homogeneous.

### Structured environments and antibiotics decrease predictability of phage-bacteria coevolution

**Figure 4:**
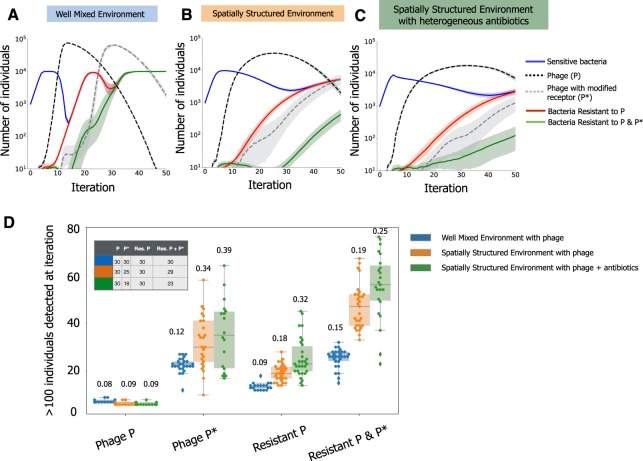
Co-evolutionary arms-race between bacteria and phage across different environments. In these simulations, bacteria can only become resistant to phage by mutating its receptor. Accordingly, phage can re-infect bacteria with a mutated receptor phenotype by mutating its own receptor. Sensitive bacteria (full blue line) are co-inoculated with virulent phages (dashed black line). Mutations conferring resistance to a random phage receptor phenotype occur at a rate of 10^−3^ per generation, and can give rise to bacterial mutants resistant to one (full red line) or two (full green line) phage receptor phenotypes. Mutations randomly changing the attachment morphology of phage P occur at 10^−4^, and can give rise to a phage with a different attachment phenotype (P*, dashed grey line). Opaque bands indicate the 95% confidence interval. (**A**) Simulations in well-mixed environments. (**B**) Simulations in spatially structured environments. (**C**) Simulations in spatially structured environments supplemented with antibiotics, that are applied heterogeneously. Here antibiotic resistant mutants emerge with probability 10^−3^. (**D**) The distribution of the iteration, in replicate simulations, at which given phage or bacterial genotypes were detected in at least 100 individuals, for the different treatment regimes in (**A–C**). Each point indicates a different simulation. The number of replicate simulations (out of 30) where the genotypes were not detected until the final time point (80 iterations) are not included in the distributions. Instead, the inset table shows the number of simulations where each phenotype has emerged in the different conditions (e.g., counter-resistance phage P* emerged only in 18 out of 30 replicate simulations performed in spatially structured environments complemented with heterogeneous antibiotics). Numbers above each distribution indicates the respective coefficient of variance, calculated based only on the simulations where the respective genotype was detected. The complete set of parameters used is shown in Supplementary Data 1.

The ability of bacteria to evolve resistance to phages might be futile if the latter adapt sufficiently fast to overcome these changes^52^. We added to the model the possibility that phages can mutate to be able to counter-act bacterial resistance. Given that phages often require multiple mutations to compensate for the evolved bacterial receptors that prevent infection^53^, we made the phage mutation rate towards novel receptors ten times lower than the mutation rate of bacteria towards phage resistance. When we allowed both bacteria and phage to evolve in well-mixed environments (Fig. 4A), we observed co-evolutionary arms races similar to both theoretical expectations^25^ and experimental observations^20^. When we imposed environmental structure, we observed slower co-evolution dynamics and higher variability between simulations (Fig. 4B). Adding antibiotics heterogeneously in the structured environments further delayed the co-evolution dynamics of bacteria and phages (Fig. 4C,D), due to the death of a significant part of the bacterial population. Like for the simulations without phage evolution (Fig. 3), surviving bacteria (either resistant to antibiotics or not exposed to lethal concentrations) were able to generate mutants resistant to phages for a longer period of time in structured environments. This is not only due to the limited diffusion of phage, but also because phages need bacterial hosts to replicate and to generate their own genetic diversity. Thus, the lower efficiency of predation in structured environments, as well as a reduction in the number of bacterial hosts due to antibiotic exposure, hinders both phage amplification and evolution.

Structured environments led to less consistent outcomes of the co-evolutionary process, compared to those taking place in well-mixed conditions (Fig. 4D). The coefficient of variation for the time of emergence of phage counter-resistance genotypes was systematically higher in structured environments, particularly when antibiotics were heterogeneously distributed (F test 0.42, P=0.013 well mixed versus structured environments; F test 0.38, P=0.01 well mixed versus structured environment with antibiotics). The coefficient of variation for the time of emergence of bacteria resistant to both phages is larger for structured environments with antibiotics, compared to well mixed environments, but non-significantly (F test, 0.65, P=0.14). However, in a significant fraction of the replicate simulations with spatial structure and antibiotics, bacteria resistant to both types of phage, as well as the mutant phage (with the counter-resistance phenotype), have not emerged at all (Fig. 4D, inset table, χ^2^ 12.6, P=0.0004 for phage with counter resistance genotype; χ^2^ 5.82, P=0.016 for bacteria resistant to both phages). When we further decrease the phage mutation rate (100x less than bacteria’s mutation rate), the phages counteracting bacterial resistance arise later, or not at all when antibiotics are heterogeneously distributed (Fig. S12). Interestingly, even in the absence of the selective pressure of these evolved phages, bacteria still acquired resistance towards them through spontaneous mutation, albeit much later in time. This further reinforces that the effect of spatial structure is much more detrimental to the generation of phage diversity, due to limited amplification, whilst promoting adaptation in bacteria. This suggests that, in natural environments, multiple stressors might render co-evolutionary arms races far less predictable than proposed by theoretical models and experimental settings that assume well-mixed environments. Together with the observation that structured environments facilitate the emergence of phage resistant bacteria (Fig. 3), this could also imply a lower success rate for combined treatments *in vivo* than suggested by *in vitro* results^46,51,54^.

### Identification of mechanisms driving invasion by lysogens

Temperate phages differ from virulent phages in their interactions with bacteria because they may become prophages and reproduce vertically with the host (lysogeny). The decision between lysis and lysogeny in our simulations mimics experimental observations: lysogeny is more frequent under high local viral concentrations because of the known mechanisms where high local phage quorum^55^ and high multiplicity of infection^56^ increase the rate of lysogenization (Fig. S13, see also Text S1). Lysogens are protected from further infections by similar phages (superinfection exclusion), leading to the decrease of free phages due to the lack of susceptible hosts (depending on phage half-life). This means that when a lysogen invader arrives at a community with resident bacteria sensitive to its prophage, lysis of a small fraction of the invaders can dramatically reduce the population of resident sensitive bacteria. This liberates resources for the lysogenic invaders^13^. This mechanism could facilitate colonization of environments by lysogens and might be harnessed to replace strains in human microbiomes. We thus studied it using eVIVALDI. The results for non-structured environments recapitulate the previous experimental data and theoretical work on this process^17,43^ (Fig. S14): prophage induction in an invader subpopulation rapidly decreases the sensitive population of residents providing a competitive advantage to colonization, but lysogenization of the latter eventually neutralizes this process (because resident lysogens are resistant to the phage).

**Figure 5:**
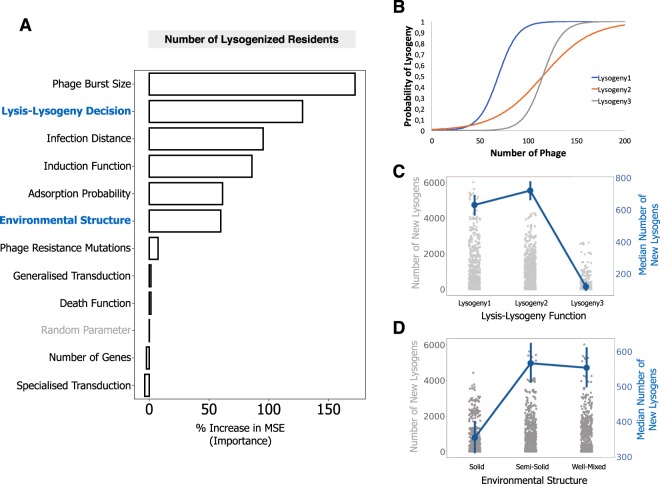
Identification of the main mechanisms affecting the generation of new lysogens using Random Forest Analysis. (**A**) An invader lysogenic population is co-inoculated in a 1:10 ratio with a resident population that is sensitive to the phage. Analysis is based on 3000 randomized combinations of parameter values and 30 repeated simulations for each combination. Parameters with a higher % in increased minimum square error have a higher importance for the measured outcome: the number of lysogenized resident bacteria at an intermediate time point (iteration 15). A random parameter (in grey) was included in the analysis to provide a baseline reference of importance. The effects of parameters highlighted in blue are detailed in panels C and D. (**B**) The three different lysogeny functions assessed with the Random Forest Analysis methodology. (**C**,**D**) The directionality of the impact of two parameters is assessed by plotting the frequency of the invader population at the end of the simulation (across all simulations), in function of the parameter of interest. In the left y-axis, and as strip plot of grey dots, is the distribution of the frequency of the invader population in all simulations. In the right y-axis, and as red dots and lines, is the median of this frequency across the simulations. The complete set of parameters used is shown in Supplementary Data 1, and parameters varied in the RFA are shown in Supplementary File S1).

Understanding the effect of lysogeny in microbial communities involves integrating several mechanisms (e.g., lysis-lysogeny decision, induction of prophages and predation itself). It is thus not straightforward to identify which parameters are the most important in a given ecological scenario. We explored a vast parameter space created by thousands of random combinations for the different parameters and analyzed how they affected the number of lysogens in the resident population at an intermediate time point (at 15 iterations, Fig. 5A). This comprehensive set of simulations showed that even if variations in the parameters affect the community outcome, most parameter combinations lead to colonization by invaders at high frequency and sometimes extinction of residents (Fig. S15).

We analyzed this vast number of simulations using a machine learning approach, Random Forest Analysis^57,58^ (RFA, see Methods and File S1), to measure the importance of the different parameters in the number of new lysogens. Our analysis shows that one of the most important mechanisms driving the lysogenization of resident bacteria is the lysis-lysogeny decision (128% increase in MSE, Fig. 5A–C). We then tested the effect of three different shapes for the lysis-lysogeny decision on the outcome of the simulation. When the number of phage particles required to produce a significant number of lysogens is very high (Lysogeny3 in Fig. 5B), few new lysogens are created (Fig. 5C) and colonization by invaders is very efficient. The two other functions (Lysogeny1 and Lysogeny2 in Fig. 5B) show similar numbers of new lysogens (Fig. 5C), suggesting that the dynamics are driven by the phage density required to generate new lysogens with a low but significant probability (~1–10%), and less by the density necessary to generate lysogens at high frequencies (i.e., close to 100%, when the curves of the lysis-lysogeny function saturate, Fig. 5B).

The overall efficiency of phage infection - given by their burst size (172%), minimum distance to infection (95%) and efficiency of adsorption (61%) – is important as well in the generation of new lysogens. Finally, the environmental structure plays also a key role in the formation of new lysogens (60%), with highly structured environments leading to a lower number of new lysogens at the intermediate point measured, compared to well-mixed, or intermediately structured environments (where the bacterial cells are fixed but their offspring can emerge within Moore distance of 3, see Methods and Text S1). Interestingly, spatial structure has previously been shown to be an important component of phage evolution, by favouring phages with a vertical mode of viral transmission^59^. This supports our inference of spatial structure as a key component in the dynamics of lysogenization of a bacterial population.

Pioneering models on the process of colonization of an environment of sensitive bacteria by lysogens were phenomenogical^43^, i.e., they were consistent with theory, but did not search to be grounded on specific mechanisms. Our analysis allows to connect directly the outcome of the model, here lysogeny of resident bacteria, to the biological mechanisms that rule this process. The combined use of RFA and eVIVALDI simulations further allows to infer, without *a priori* assumptions, the importance of different mechanisms on the outcome. This allows the design of experiments focusing on the effect of these major mechanisms on the processes of interest. It also provides a way of choosing, from all the possible mechanisms, those that can be modelled in more detail using classical approaches.

### The efficiency of phage induction as a competitive strategy is modulated by the environmental structure

Given the importance of environmental structure in the dynamics of lysogeny, it is expected that *in vivo* experiments might lead to observations that are harder to capture assuming well-mixed environments. The advantage of lysogens in the colonization of the mouse gut inhabited by resident sensitive bacteria was recently demonstrated experimentally^21^. In this pioneering study, it was suggested that the efficiency of invasion depends on the initial ratio between invaders and resident cells. Indeed, our simulations considering different initial ratios of invading lysogens versus resident non-lysogens are in agreement with the previous experimental and theoretical results, showing that the latter were more likely to survive as lysogens when more abundant in the beginning of the process (Fig. S16). The abovementioned study presented a population-based deterministic mathematical model that fitted well most experimental data, but predicted faster initial infection rates than the observed ones. While different parameters can slow down these dynamics (e.g., the burst size of the phage^21^), the spatially structured mouse gastrointestinal tract is likely to interfere with the temporal dynamics of lysogeny, as suggested by the RFA.

**Figure 6:**
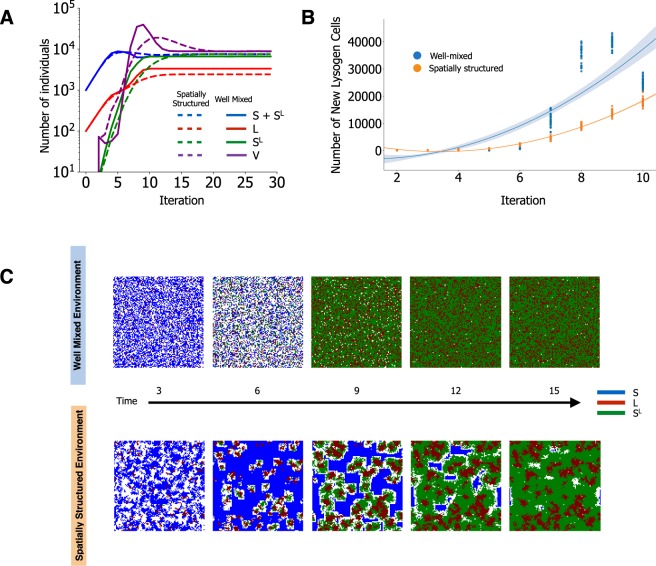
Environmental structure impacts the dynamics of lysogen colonization. (**A**) Invading lysogens (L, red lines) and resident sensitive cells (S, blue lines) are co-inoculated at a ratio of 1:10. Phages (purple lines) are spontaneously induced and generate new lysogens in the sensitive resident cells (S^L^, green lines). Full lines: well-mixed environments. Dashed lines: spatially structured environment. Data was displayed as in Fig. 3 of ^21^ for comparison. (**B**) Emergence of resident lysogens in well-mixed (blue) and in spatially structured (orange) environments during the initial 10 iterations of the simulations shown in B. Shown is the polynomial fit of order 2 for the initial 10 iterations, for each of the two types of environment; ANCOVA between the two environments, F = 485.5, p = 0. (**C**) Spatial organization for a representative simulation in well-mixed (top) or structured (bottom) environments. The complete set of parameters for these simulations is show in Supplementary Data 1.

We observed several differences in the population dynamics of invaders, residents and phages between simulations with structured and well-mixed environments. Notably, structured environments led to smaller final populations of invading lysogens (Fig. 6A), but also to slower generation of lysogens in the resident strain (Fig. 6B), as noted to occur in the abovementioned study^21^. This also fits experimental data on *P. aeruginosa* where lysogenic conversion was observed at lower rates in the lung of mice than in liquid *in vitro* environments^60^. Previous theoretical studies postulated that spatially structured environments have an effect in the process of lysogeny, for instance by increasing the frequency of multiple infections in a single cell^61^. In our simulations, phages do concentrate at higher densities faster in structured environments (where diffusion is limited) leading to a faster lysogenization of nearby residents. However, this is balanced by the limited diffusion of phage particles that decreases predation (see Fig. 2), lowering the frequency of invaders (full vs dashed red line in Fig. 6A, Fig. S17), and retarding residents’ lysogenization (Fig. 6A,B). Structured environments also limit the expansion of the initially few lysogenized residents, which face the competition of invading lysogens and non-lysogenized residents that have not yet been predated (Fig. 6B,C). Hence, structured environments affect the ability of lysogenic bacteria to invade in multiple ways. Firstly, lysogens arise more rapidly in the early stages, reducing the amplification of virions (since new lysogens are resistant to phages), a phenomenon that was previously observed to affect the epidemiology of phage infections^59^. Secondly, structure limits the diffusion of the induced prophages, slowing-down the lysogenization of the residents. Thirdly, newly lysogenized cells increase in numbers at a lower rate, due to their limited diffusion during reproduction and a lower availability of resources used by the not yet predated residents. Thus, although structured environments provide slower dynamics of new lysogenization events, they benefit resident bacteria by limiting the efficiency of invaders. Moreover, structure potentiates the coexistence of the three different subpopulations (invaders, residents and lysogenized residents) for longer periods of time (Fig. 6C).

## Conclusions

Given the complexity of *in vivo* systems, models are often better suited to identify qualitative behaviors than to produce quantitatively precise predictions^19^. Individual-based models provide novel ways to analyze and predict the behavior of microbial systems, being well positioned to account for the complexity of mechanisms, environments and agents present in *in vivo* contexts^36^. The present version of the eVIVALDI model integrates multiple bacterial species, different types of phages, and environmental structure. This allowed us to unravel temporal changes in community composition caused by antibiotics and/or phages. Nevertheless, IBMs, like all models, have limitations and use simplifying assumptions. Some of them, arise from their complexity. IBMs require a large number of parameters to represent complex biological mechanisms and this complicates the identification of the most important ones. We used RFA to understand how changes in parameters affected the model, and which parameters were more relevant. This allowed the selection of variables for more detailed study. Albeit this strategy can be used with other modelling approaches, IBMs can, in principle, include mechanisms and parameters that are empirically similar to those acting on individual biological entities, increasing the usefulness of RFA for experimenters. Yet, further work will be needed to understand the relevance of minor parameters in these processes. An additional complication arises from the focus of IBMs on individuals when many of these parameters have been quantified at the level of populations. This forced us to make assumptions of how to translate populational to individual parameters, and simulations to illustrate how the two are associated (e.g., Fig. S5). This problem is likely to become less important in the future. Emerging single-cell technologies provide individual-based estimates and will probably spur the development of IBMs. Finally, the computational costs of IBMs led us to follow fewer cells than typically used in experimental settings (although which of the two best represents *in vivo* setups is subject to discussion). This was done to decrease the computational time of the simulations, but may increase the effect of drift and did require the increase of certain values (e.g., mutation rates) to increase the probability of detecting the corresponding events. We repeated some key simulations with higher population sizes and this revealed similar qualitative dynamics (Figs S18 and S19). Hence, we believe this does not affect the major results of our study.

eVIVALDI encapsulates a large number of parameters, but some are still missing. We do not yet model temporal or spatial variations of environmental structure, which may limit the emergence of population structure (not a focus of this study). Also, we do not yet model bacterial physiology and how it affects phage infection or the efficiency of antibiotics. In our simulations, the bacteria are actively growing and the census population sizes are stable (in the absence of antibiotics and phages). These are conditions favorable to phage infection and should be representative of environments like the human gut. Future work should consider changes in bacterial physiology and how they affect the system (e.g., the effect of receptor’s expression or growth rate^62–64^). Despite these limitations, our model systematically produced behaviors consistent with previous experimental and theoretical observations when they were available. Additionally, a more thorough robustness analysis for some of our key experiments (see Figs S20 and S21) confirms that our results remain consistent, even when there is a significant amount of noise in the initial parameters. Nevertheless, we expect that eVIVALDI can be used, improved and enriched by the research community to include more complete and accurate mechanisms. An advantage of IBMs is that they are easily extensible, and therefore the improvement of the model can be done at the community level since we share our code in a Git system.

Our model showed the importance of structured environments in phage-bacteria interactions. The major effect of structured environments, limited phage diffusion, hinders the phages’ predation efficiency and amplification. This fits a number of theoretical works on host-pathogen interactions in structured environments, where the spread of highly transmissible pathogens is impeded by local depletion of susceptible hosts (“self-shading”)^65^. Environmental structure also increases the rate of acquisition of mutations that provide resistance to both antibiotics and phage. This is concomitant with a decreased ability of the phage to acquire counter-mutations, as a consequence of their limited amplification in such environments. This is particularly prevalent when antibiotics are not homogeneously distributed, as seems common in natural settings^49,50^. Together, this suggests that structured environments lead to arms-races dynamics that are slower and less predictable, since phages can sometimes become extinct before acquiring adaptive mutations, and where single resistances are acquired slower, but multiple resistances are acquired faster.

The use of RFA allowed to highlight the most important mechanisms underlying the observed dynamics. Most of these concerned characteristics of the phage itself, including its burst size and the regulation of the entry on the lytic cycle. Hence, the success of a lysogen in displacing a given strain depends a lot on the characteristics of the induced prophage, while environmental structure is the most important factor that is not associated with the phage itself. The effect of environmental structure had not been modelled, to the best of our knowledge, on this peculiar system where competition between groups takes place by the use of a parasite for which invaders are immune and for which sensitive resident individuals can also become immune. The detailed study of this variable showed that the dynamics of lysogeny are slower in structured environments, but that this is more than compensated by the lower colonization efficiency of invading lysogens. As such, different bacterial populations (invaders, residents and lysogenized residents) are expected to coexist for longer periods of time, compared to well-mixed environments. Importantly, the success of the lysogen in displacing the resident sensitive bacteria seems to decrease with environmental structure, *i.e*., it renders the process of colonization less efficient.

Overall, we have shown that environmental structure directly impacts the dynamics of phage-bacteria interactions. The use of IBMs provides a method to associate these results with molecular mechanisms for subsequent experimental test. Hence, these results should be pertinent to improve the design of phage therapies and to understand the impact of phages in microbial systems.

## Supplementary information

Supplementary Information

Video S1

File S1

Table S1

Dataset 1

## Data Availability

The source code of the eVIVALDI model is available for download at the following GIT repository: https://gitlab.pasteur.fr/jsousa/eVIVALDI. The input files used to simulate the data in the main manuscript are available as supplementary data. Other input files, the output files of all the simulations and the scripts used to generate the figures are available from the corresponding author upon request.
