# Supplementary material for: Environmental structure drives resistance to phages and antibiotics during phage therapy and to invading lysogens during colonisation
Source: Sci Rep. 2019 Feb 28;9:3149. doi: 10.1038/s41598-019-39773-3 (PMC6395636; doi:10.1038/s41598-019-39773-3)
Supplement: Supplementary file 1 — Supplementary Information [file 41598_2019_39773_MOESM1_ESM.pdf]

Supplementary Information for:

Environmental structure drives resistance to phages and antibiotics during phage therapy and to invading lysogens during colonisation

Jorge A. Moura de Sousa[1,2,*] , Eduardo P.C. Rocha[1,2]

[1]Microbial Evolutionary Genomics, Institut Pasteur, 28, rue Dr Roux, Paris, 75015, France. [2]CNRS, UMR3525, 28, rue Dr Roux, Paris, 75015, France.

* corresponding author: jorge-andre.sousa@pasteur.fr

Table of contents

Supplementary Figure 1 .... **3**
Supplementary Figure 2 .... **4**
Supplementary Figure 3 .... **5**
Supplementary Figure 4 .... **6**
Supplementary Figure 5 .... **7**
Supplementary Figure 6 .... **8**
Supplementary Figure 7 .... **9**
Supplementary Figure 8 .... **10**
Supplementary Figure 9 .... **11**
Legend Supplementary Video 1 .... **12**
Supplementary Figure 10 .... **13**
Supplementary Figure 11 .... **14**
Supplementary Figure 12 .... **15**
Supplementary Figure 13 .... **16**
Supplementary Figure 14 .... **17**
Supplementary Figure 15 .... **18**
Supplementary Figure 16 .... **19**
Supplementary Figure 17 .... **20**
Supplementary Figure 18 .... **21**
Supplementary Figure 19 .... **22**
Supplementary Figure 20 .... **23**
Supplementary Figure 21 .... **24**
Supplementary Text S1 (ODD) **25**
References for Text S1 (ODD) **47**

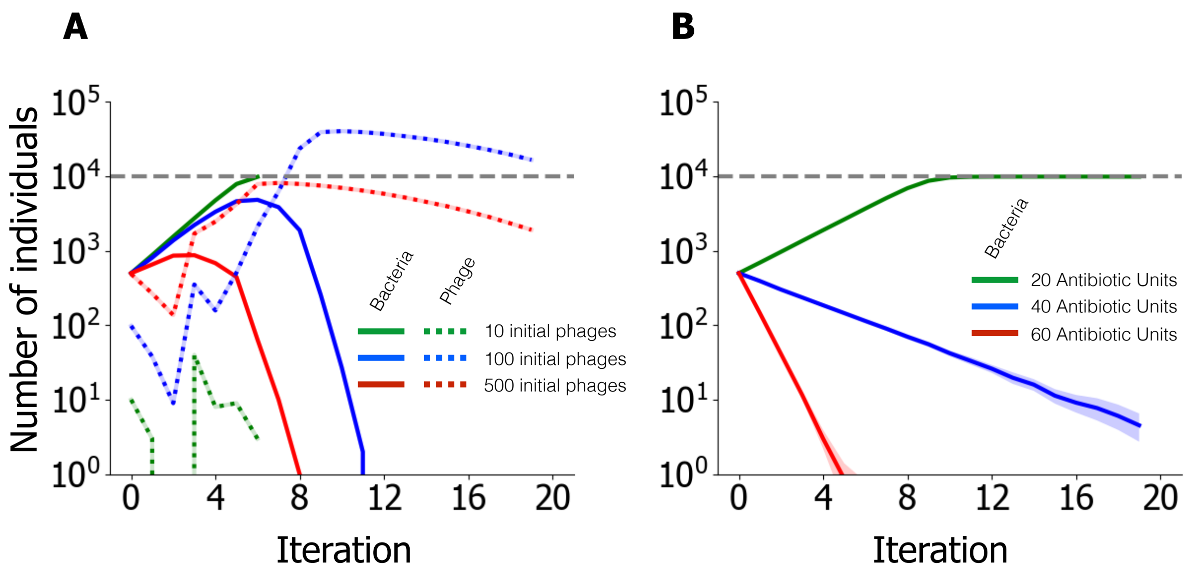

SUPPLEMENTARY FIGURE 1 – Short term dynamics of different phage or antibiotic loads from the perspective of an infection. A) Phages are inoculated at different loads, and the populations of bacteria (full lines) and phages (dotted lines) are followed. The threshold of an illustrative infection (i.e., the number of bacteria that leads to host death) is indicated by the grey dashed line. Lines show the output of a single representative simulation. B) Antibiotics are applied at different concentrations. Note that the dynamics of action for antibiotics differ from the work of Levin and Bull (ref 31 in the main text) because in our simulations antibiotics have an effect on bacterial mortality regardless of the physiological state of the cell (See Text S1). Simulations in both A and B are performed in well-mixed conditions. The simulation(s) were truncated when the infection threshold was reached for the lower phage load (green line in A).

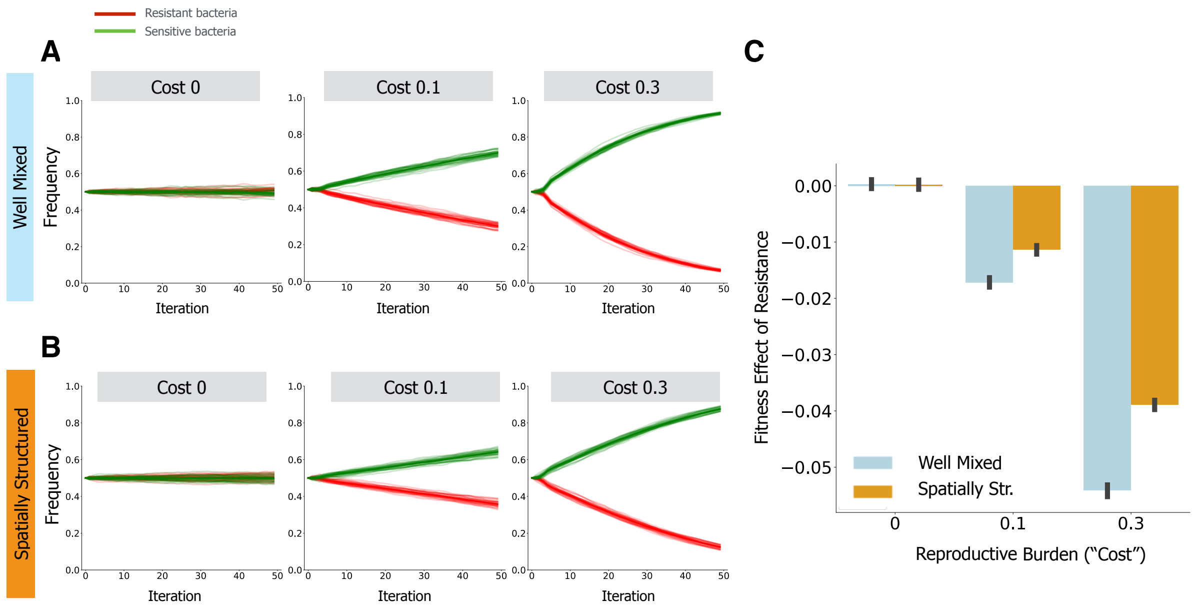

SUPPLEMENTARY FIGURE 2 – **The costs of resistance.** The effect of the cost parameter is followed in terms of reproduction of individual cells with a costly trait (red) while in competition with cells without a costly trait (green). **A)** the subpopulation dynamics in well-mixed environments, for different costs. **B)** The same as in **A**, but with spatially structured environments. **C)** the fitness effect of the costly trait is calculated from the population changes in **A** and **B** over 20 iterations, according to (LN(freq_resistant_final)-LN(freq_resistant_inital))/(final_time – initial_time).

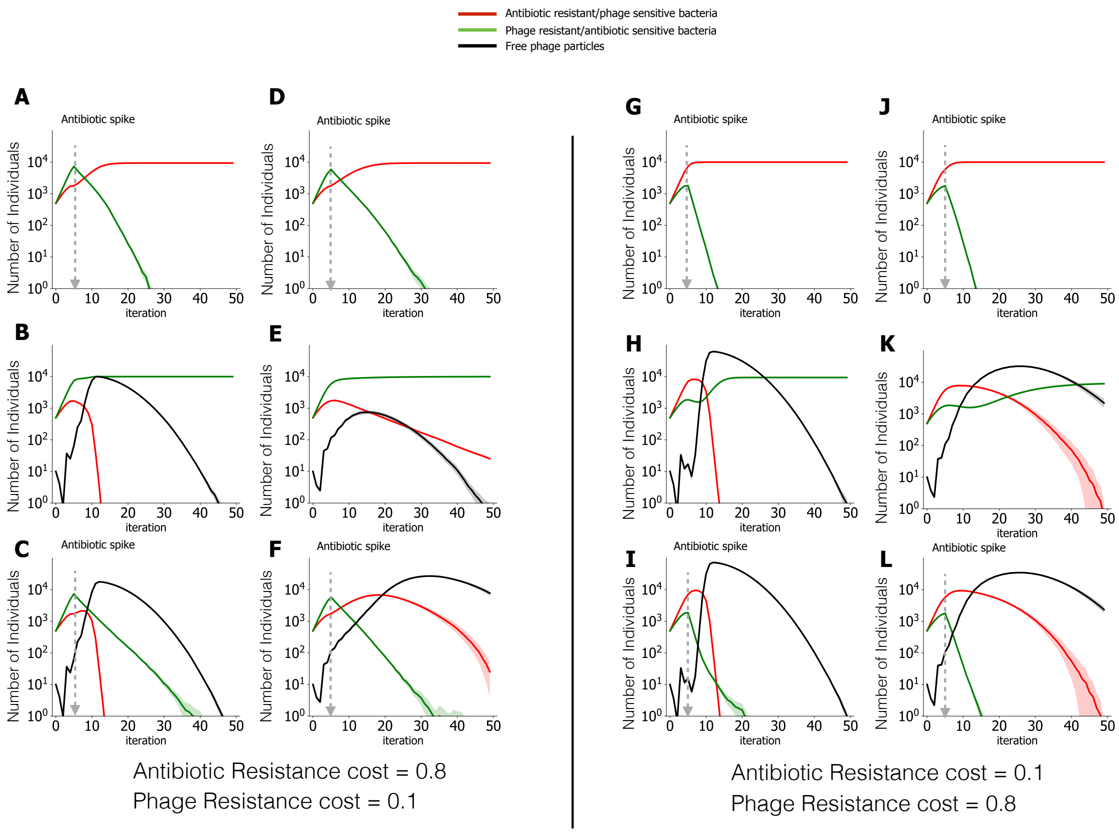

SUPPLEMENTARY FIGURE 3 – **Effect of costs of resistances in population dynamics.** Conditions of simulations and representation of data are similar to the ones simulated for Figure 2 in the main text, but with the cost of phage resistance increased to 0.8 (A-F), or the cost of antibiotics resistance increased to 0.8 (G-L).

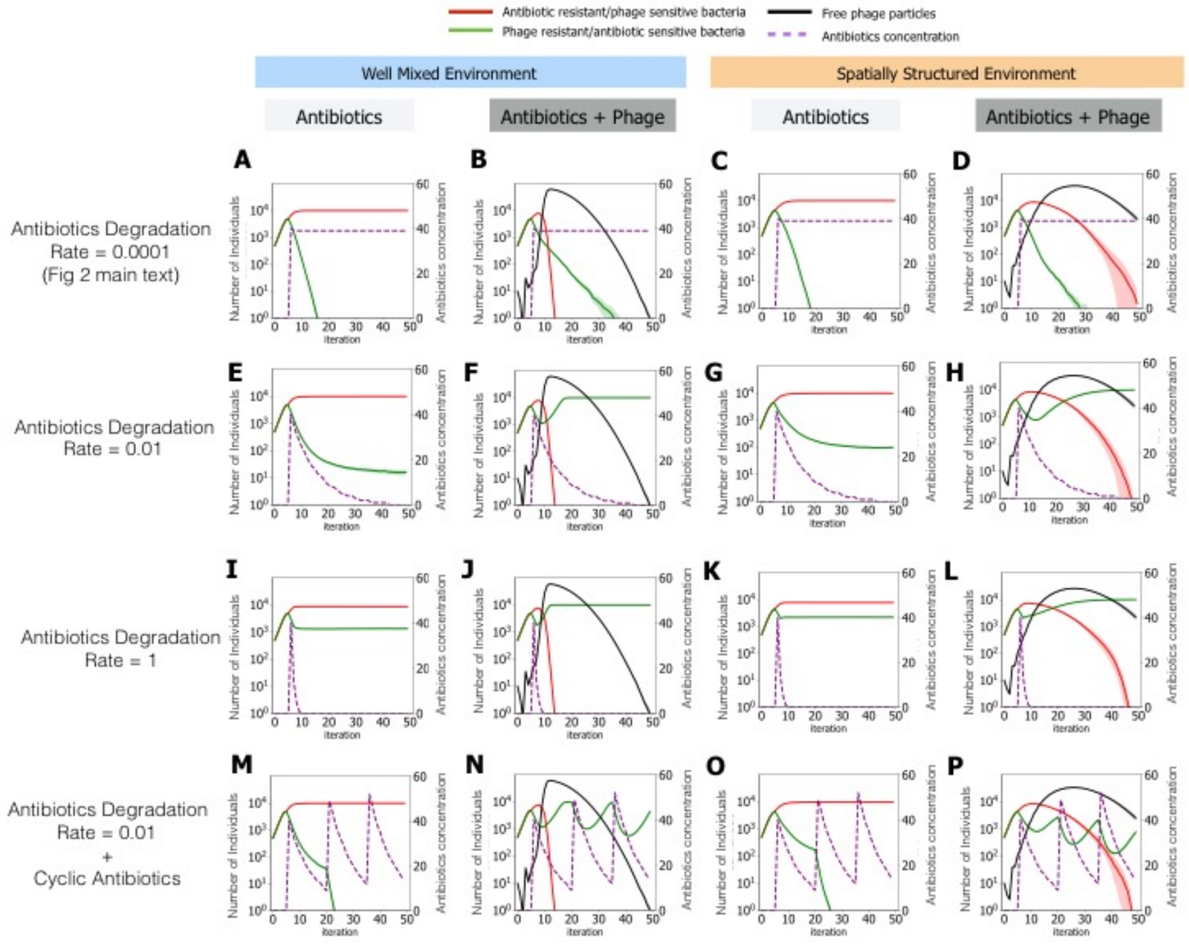

SUPPLEMENTARY FIGURE 4 – Effect of different antibiotics pharmacokinetics and treatment regimes in population dynamics. Conditions of simulations and representation of data are similar to the ones simulated for Figure 2 in the main text, with the addition of dashed purple lines that show the antibiotics concentration over time (secondary y-axis). Shown are only simulations with antibiotics only (A, C, D, E, G, I, K, M, O) or with antibiotics and phages (B, D, F, H, J, L, N, P). In panels (A-D), antibiotics degradation rate is similar to the ones in Figure 2 of the main text (0.0001). In panels (E-H), antibiotics degradation rate was increased to 0.01, and in panels (I-L) antibiotics degradation rate was increased even further to 1. In panels (M-P), antibiotics degradation rate is 0.01, but additional antibiotics doses are added at iterations 20 and 35, besides the initial spike at iteration 5.

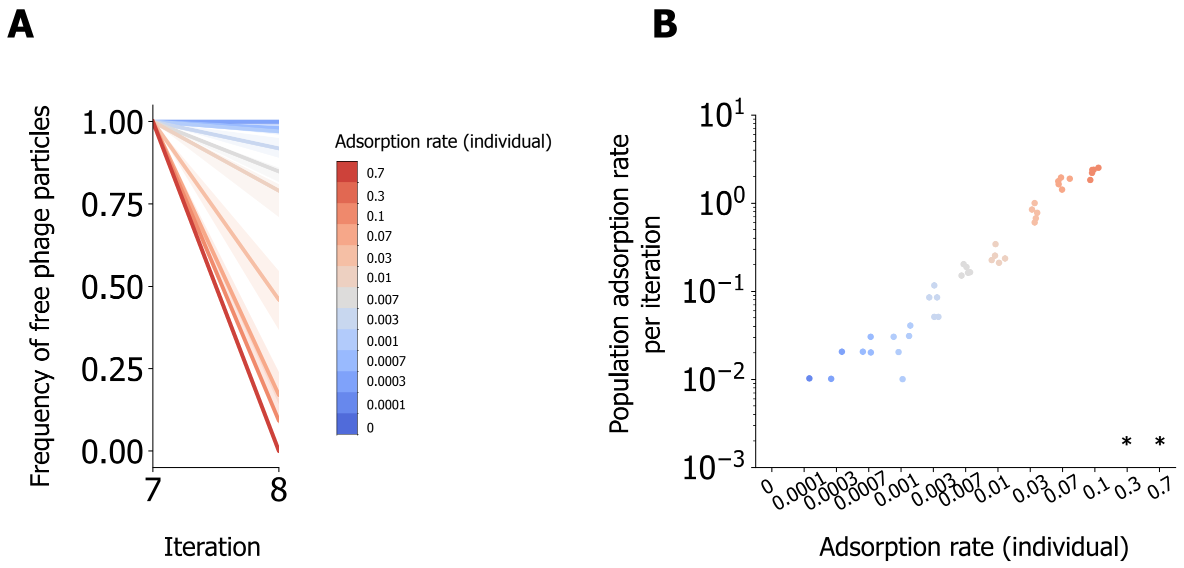

SUPPLEMENTARY FIGURE 5 – **Relationship between individual and population-level rates of phage adsorption rates. A)** Frequency of free phage particles (i.e., not currently infecting a bacterium) over the total number of phage particles (infecting and non-infecting) over one iteration, for different values for the adsorption rate (probability) of a single phage particle upon encountering a bacterium. Lines show median of 5 simulations, with shaded areas showing 95% confidence interval. **B)** Calculation of the population-wide adsorption rate per iteration (alpha), where alpha=ln(Ntotal/Nfree), with simulations that start with an initial period of bacterial growth, until 10^4, and the addition of 10^2 phage particles. Population adsorption rate is calculated in the iteration immediately following addition of phages to the environment. Dots show the population adsorption rate for 5 replicate simulations with different values of individual phage particles adsorption rate. Population adsorption rate below 10^{-3} were deemed undetected, and those replicates are not show. Likewise, high values of individual adsorption rates (0.3 and 0.7) lead to no free phages after one iteration, and thus the calculation of the population adsorption rate is not possible.

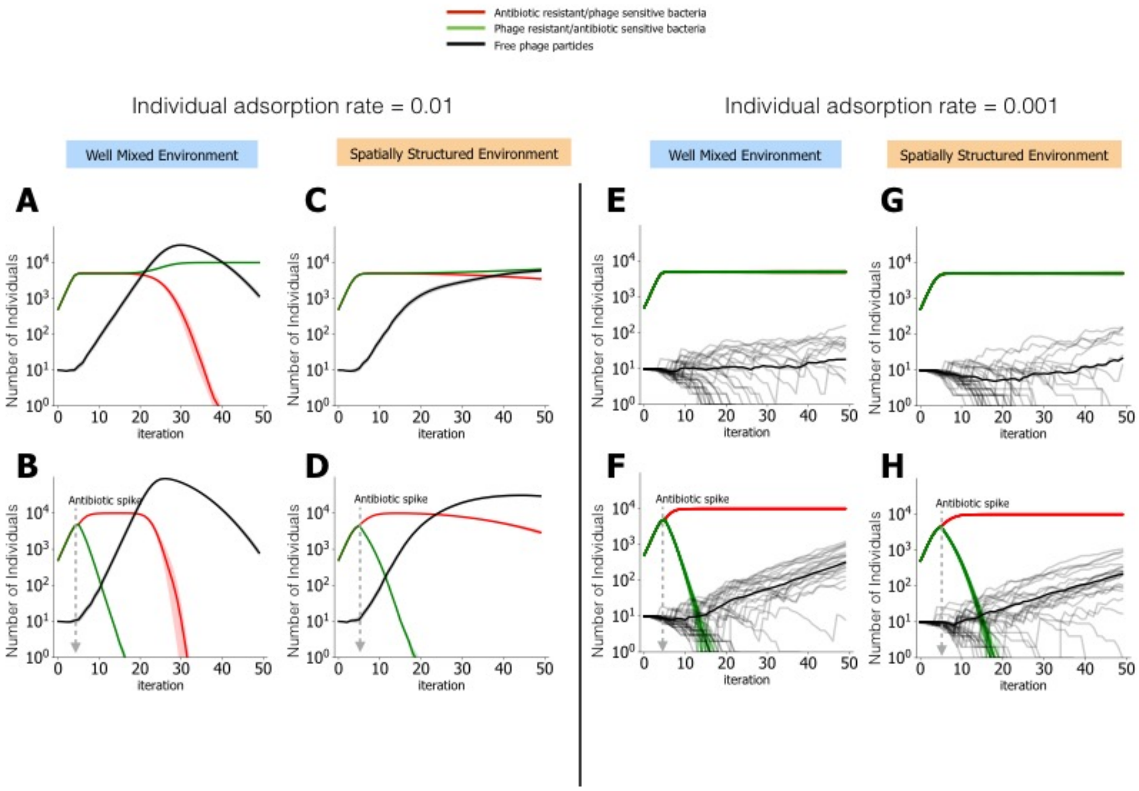

SUPPLEMENTARY FIGURE 6 – **Effect of adsorption rate in population dynamics.** Conditions of simulations and representation of data are similar to the ones simulated for Figure 2 in the main text, but with an individual phage adsorption rate of 0.01 (A-D) or 0.001 (E-G). Show are simulations when phages only (A,C,E,G) or both antibiotics and phages (B, D, F, H) are used, either in well-mixed environments (A, B, C, D) or structured environments (E, F, G, H). Panels A-D show shaded areas as 95% confidence interval, as in Figure 2, but panels E-H show the individual replicate simulations explicitly, so that the extinctions of the phage populations can be appreciated.

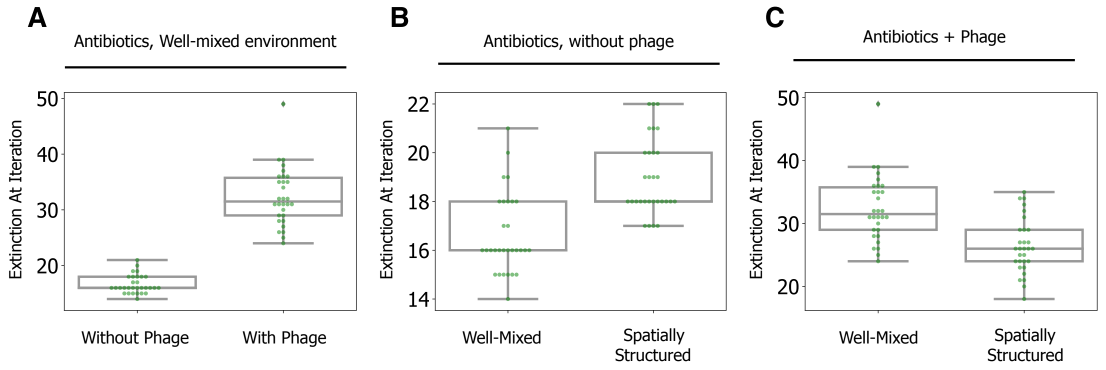

SUPPLEMENTARY FIGURE 7 – **Extinction of antibiotic sensitive population is modulated by the existence of phage and spatial structure.** Distributions of the final iteration (y-axis) in which antibiotic sensitive bacteria are detected. **A)** Effect of the presence of bacteriophage in well-mixed environments. **B)** Effect of spatial structure in an antibiotic-only regime. **C)** Effect of spatial structure in a selective regime of antibiotics and phage.

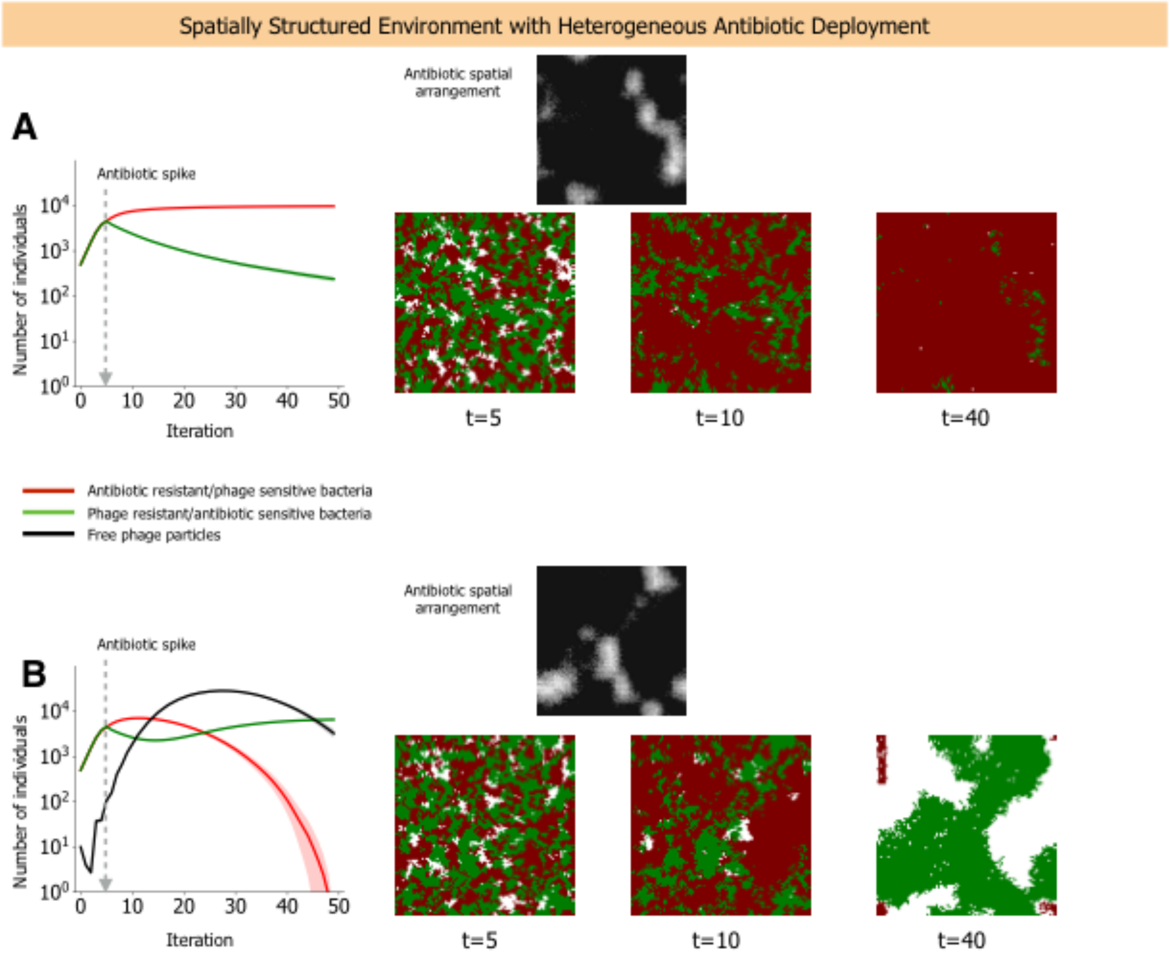

SUPPLEMENTARY FIGURE 8 – **Heterogeneous antibiotic exposure leads to co-existence between sensitive and resistant strains.** Antibiotics are applied heterogeneously in spatially structured environments (contrary to the homogeneous antibiotic exposure in Figure 2d-f), with some regions having more antibiotic concentration than others (respectively, darker and lighter areas in the "Antibiotic spatial arrangement" panels). In **A)**, antibiotics are applied at the indicated time. In **B)**, antibiotics are applied at the indicated time and virulent phages (10 particles) are co-inoculated with the bacteria at time 0. In front of plots with the population dynamics is a representative time lapse at 3 time points of the lattices in each scenario, where the colors represent the bacterial species and white spaces represent the absence of bacterial cells.

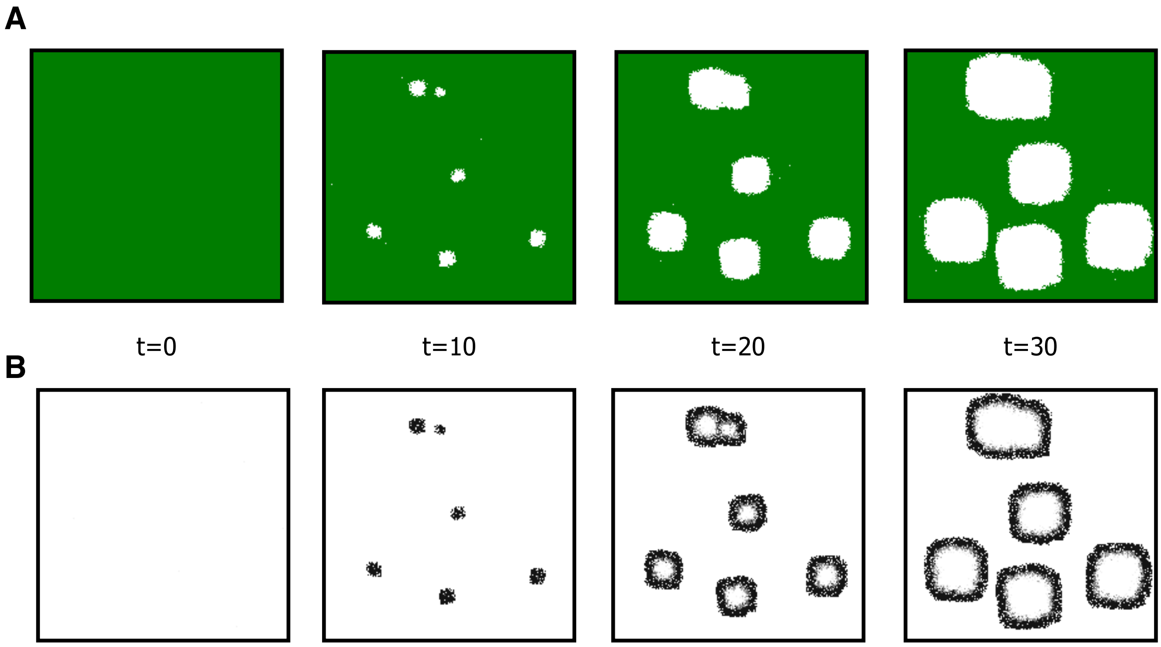

SUPPLEMENTARY FIGURE 9 – **Spatial structure creates local predation and the formation of phage plaques.** A spatially structured environment with a single bacterial species (in green) is inoculated with 6 phages randomly located across the environment. In A), the time-lapsed interval of the bacterial cells, where white spaces indicate the absence of bacteria. In B), the same time-lapsed interval, with a different view of the environment, with phage shown as grey cells, and darker cells indicated a larger phage concentration in that location. The full dynamics can be seen in Supplementary Video 1.

SUPPLEMENTARY VIDEO 1 – **Temporal dynamics of phage predation and plaque formation.** Full temporal dynamics of the time-interval snapshots shown in Supplementary Figure 4. The first two panels show information as explained in Supplementary Figure 9. The third, rightmost panel indicates (in blue) the bacterial cells that are infected by phage at each instant.

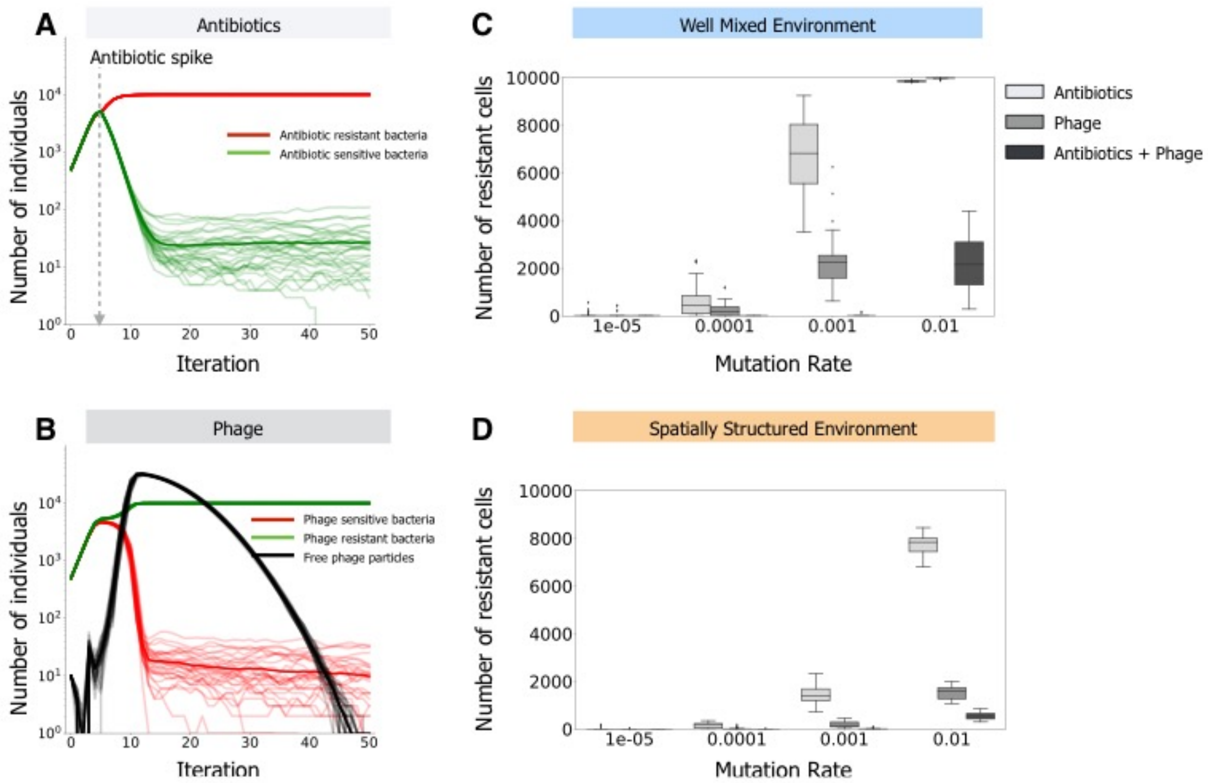

SUPPLEMENTARY FIGURE 10 – **Community dynamics when resistance to antibiotics and/or virulent phage can evolve.** A) and B) Ecological setup is similar to the ones in Figures 2A and 2B (main text, respectively), but resistance to antibiotics or phages arises at a frequency of 10^{-3}. Lines show each of the 30 replicate simulations. C) and D) A population initially composed of a single species, sensitive to both antibiotics and phage, is subjected to different selective regimes, antibiotics, phage, or both in respectively well-mixed and spatially structured environments. Different mutation rates for the locus responsible for resistance to the different selective agents are shown in the x-axis. Boxplots indicate the distributions of the number of resistant mutants found in 30 realizations of the simulations after 15 iterations.

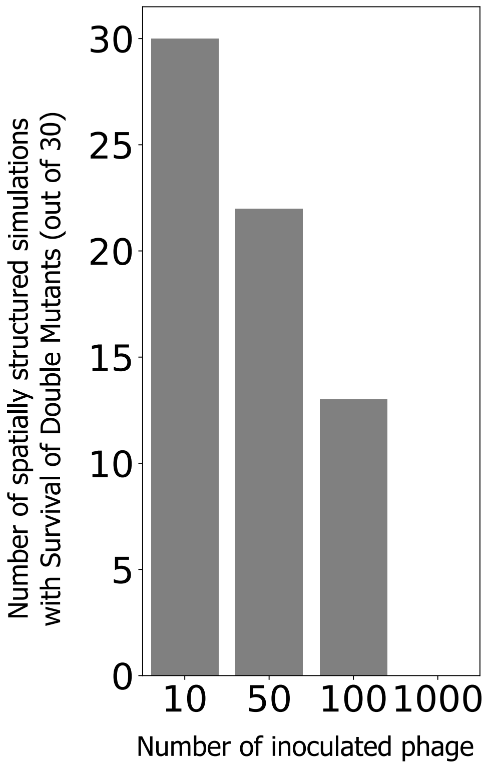

SUPPLEMENTARY FIGURE 11 – Load of co-inoculated phage modulates the emergence of multi-resistant bacteria. The effect of increasing the number of phages initially co-inoculated with bacteria (x-axis) leads to a decrease in the number of simulations where double mutants (resistant to antibiotics and phage) are observed, in spatially structured environments.

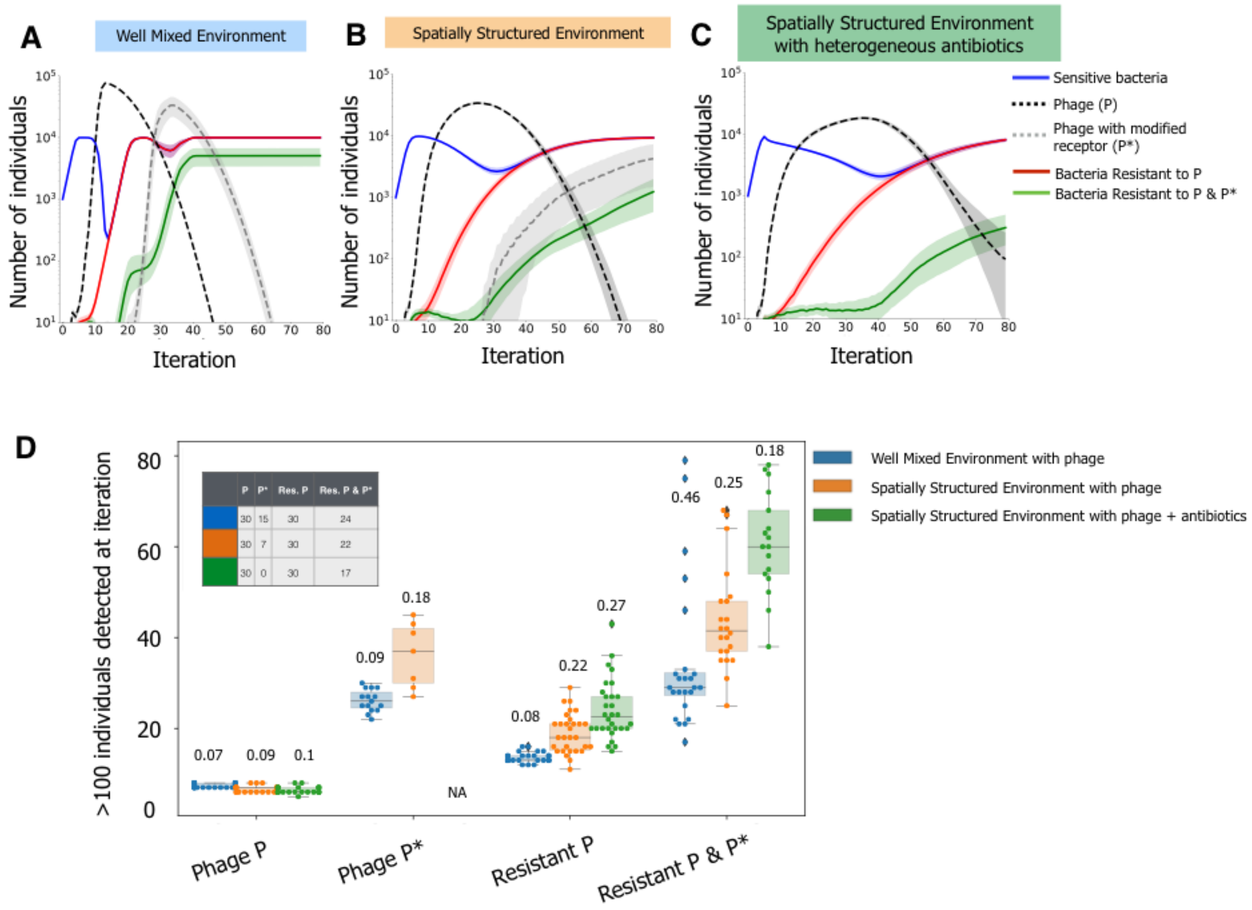

SUPPLEMENTARY FIGURE 12 – Effect of a phage mutation rate 100x lower than the bacterial mutation rate in co-evolutionary dynamics. Conditions of simulations and representation of data are similar to the ones simulated for Figure 4 in the main text, but with a phage mutation rate of 10^{-5}.

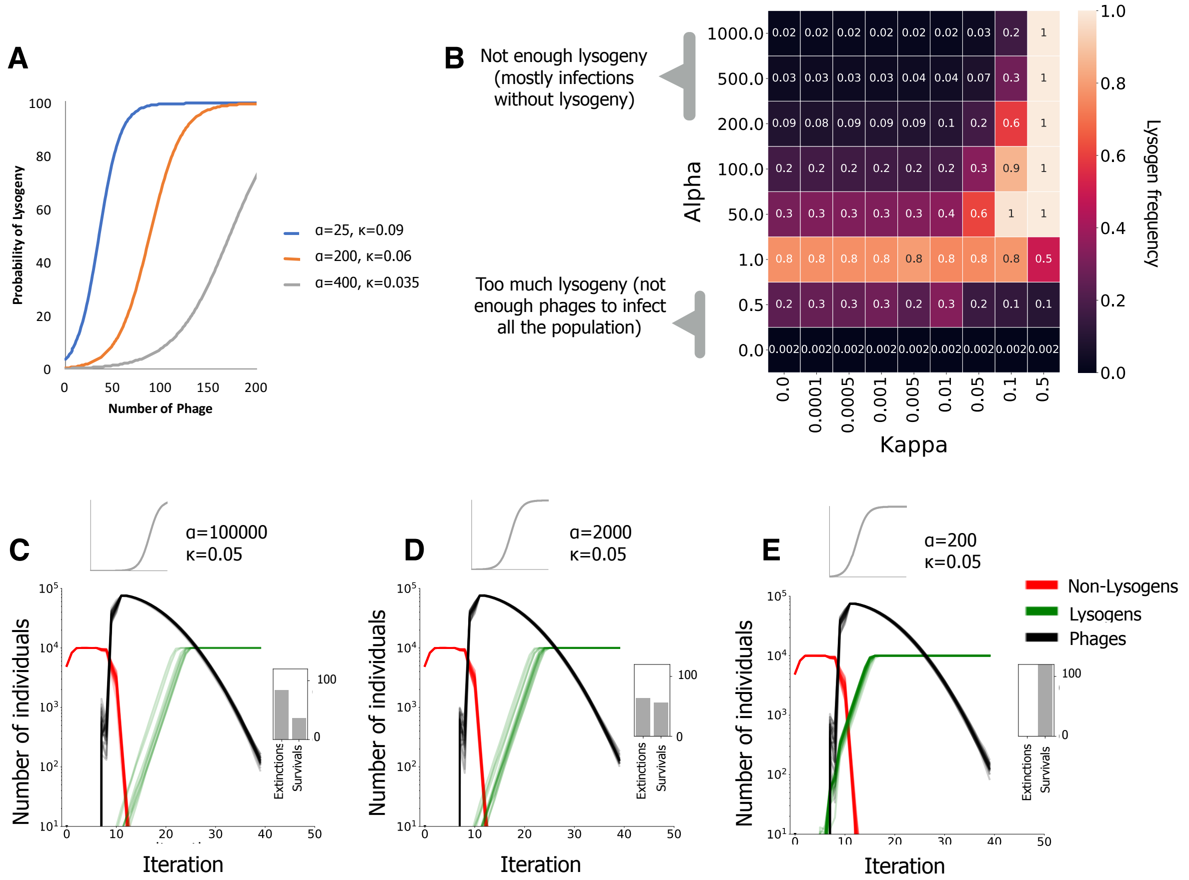

SUPPLEMENTARY FIGURE 13 – The dynamics of the simulated lysis-lysogeny decision. A) Different parameterizations of the equation determining the lysis-lysogeny decision. This decision is influenced by the number of phage surrounding the infected host (x-axis), but the function can be parameterized to generate lysogens in the absence of surrounding phage (intersect at x=0). In **B)**, a heatmap is shown with the mean frequency of lysogens (over 30 repeated simulations) detected after 12 iterations, according to different possible parameterizations of the lysis-lysogeny decision function (0 indicates no lysogens detected, 1 indicates 100% of the population is composed of lysogens). In **C-E)** a bacterial population, initially composed of bacteriophage sensitive, non–lysogenic cells, is co-inoculated with temperate phage. Red lines indicate the non-lysogenic bacterial populations, whilst green lines indicate the number of bacterial cells that have become lysogens with the phage. Black lines indicate the number of temperate phage in the environment. The different panels indicate the different functions used for the lysogeny-lysis decision by the temperate phage (parameters and curve is indicated above each panel). Lines indicate the different realizations in 30 replicate simulations. Barplots as inboxes indicate the percentage of simulations that lead to either the extinction or survival of the bacterial populations.

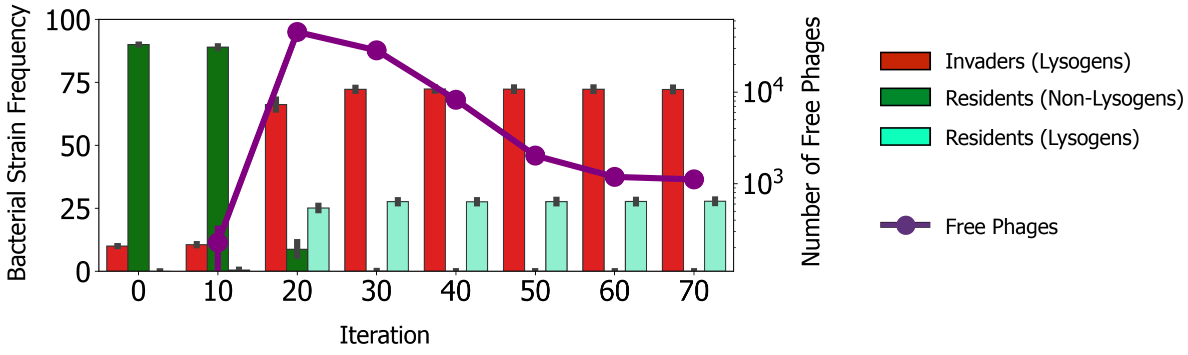

SUPPLEMENTARY FIGURE 14 –The role of lysogeny in community dynamics. Genomes from species A (invaders, red bars) carry an inducible prophage, whereas those of species B (residents, dark green bars) are initially non-lysogens. Species are co-inoculated at a 1:10 mixture. Phages (purple line) are spontaneously induced from the lysogenic population. These phages infect the sensitive resident population, which may form lysogens that are protected from phages (B*, light green bars). Eventually, the resident that are not lysogens become extinct. All bars represent the average of 30 replicate simulations with similar parameters, with the error bars indicating their 95% confidence interval. For comparison with the original experimental data, simulation results were displayed as in Figure 1 of reference 17 of the main text.

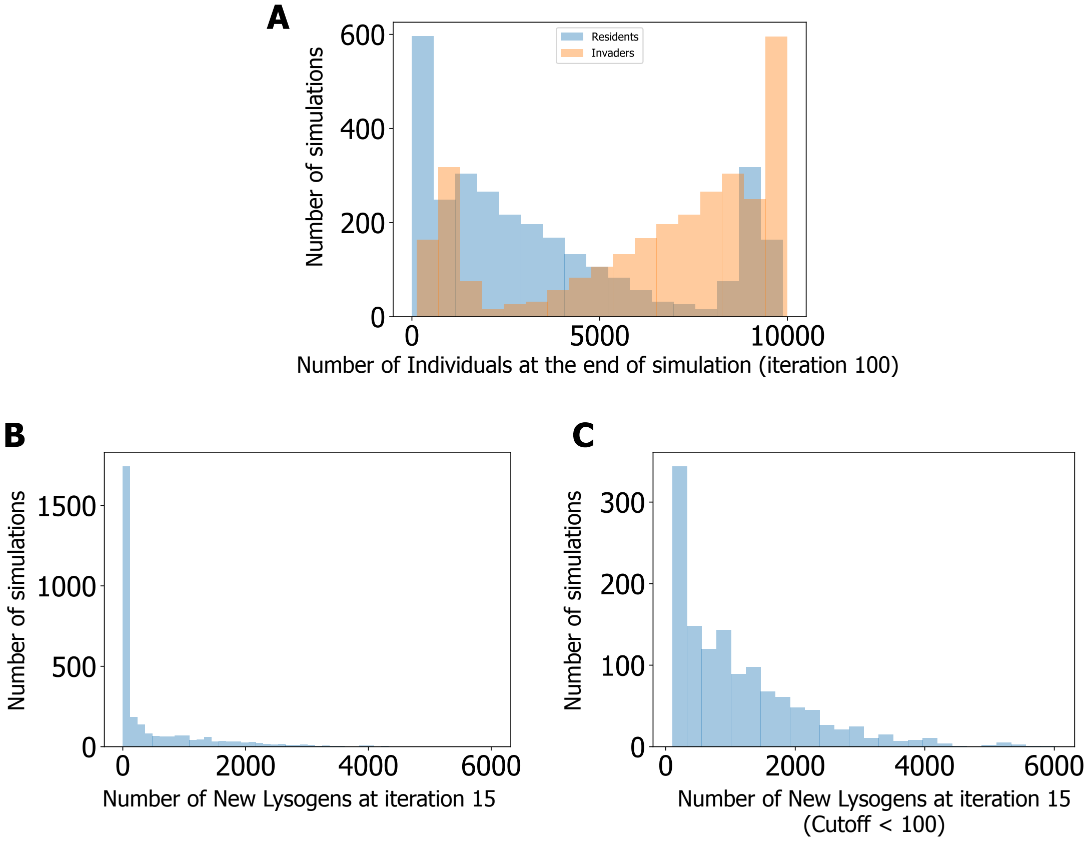

SUPPLEMENTARY FIGURE 15 – Distribution of outcomes explored with RFA. A) Distribution of the number of residents (blue) and the number of invaders (orange) in the 3000 simulations used for RFA, at the final time point (iteration 100). B) Distribution of newly lysogenized residents at iteration 15, in all 3000 simulations used for RFA. C) Same as in B, but with a cutoff for simulations with at least 100 newly lysogenized cells detected at iteration 15.

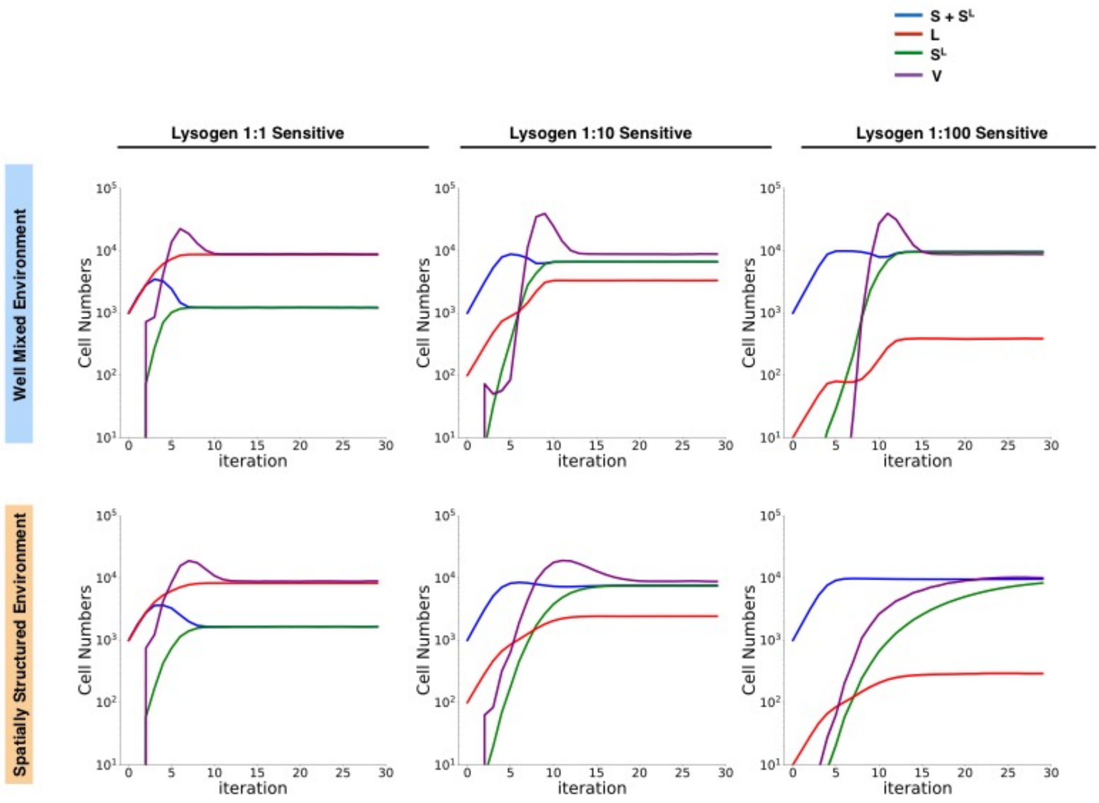

SUPPLEMENTARY FIGURE 16 – **The dynamics of lysogeny depend on the initial ratio between lysogen and non-lysogen populations.** Invaders (lysogens, red lines) and residents (non-lysogens, blue lines) cells are co-inoculated at the ratio indicated above the plots. Induction of prophage occurs intrinsically at phage are released into the environment (purple lines), where they infect and kill sensitive cells. In this process, new lysogens are formed in the resident population (green lines). The final equilibrium between lysogens and newly formed lysogens is different for the different initial ratios. Top panels indicate simulations in well-mixed environments, whilst the panels in the bottom row indicate simulations in spatially structured environments. All lines are the average of 30 repeated simulations with the shaded areas indicating 95% confidence interval.

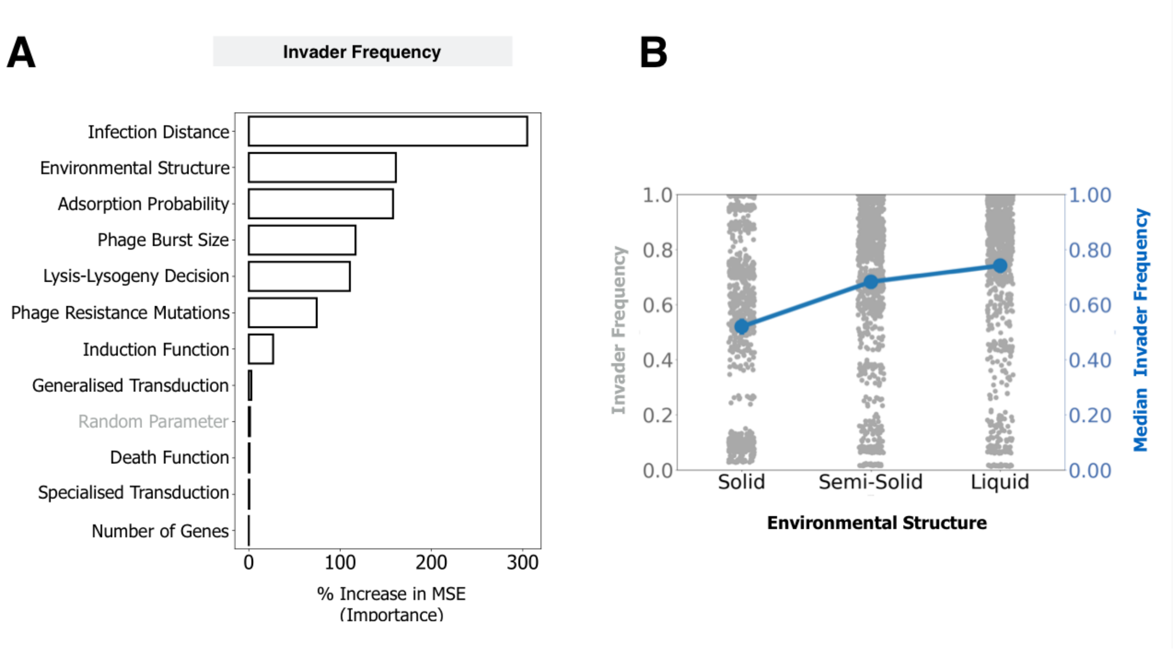

SUPPLEMENTARY FIGURE 17 – **Identification of the main mechanisms affecting the frequency of invaders at the end of the simulations. A)** Conditions and underlying simulations are the ones used in Figure 5A (main text), but the response variable used to quantify the different parameters using Random Forest Analysis is the frequency of invaders at the end of the experiment. B) The directionality of the impact of environmental structure in the frequency of invaders at the end of the simulations is assessed by plotting this across the different types of environmental structure. In the left y-axis, and as strip plot of grey dots, is the distribution of the frequency of the invader population in all simulations. In the right y-axis, and as blue dots and lines, is the median of this frequency across the simulations.

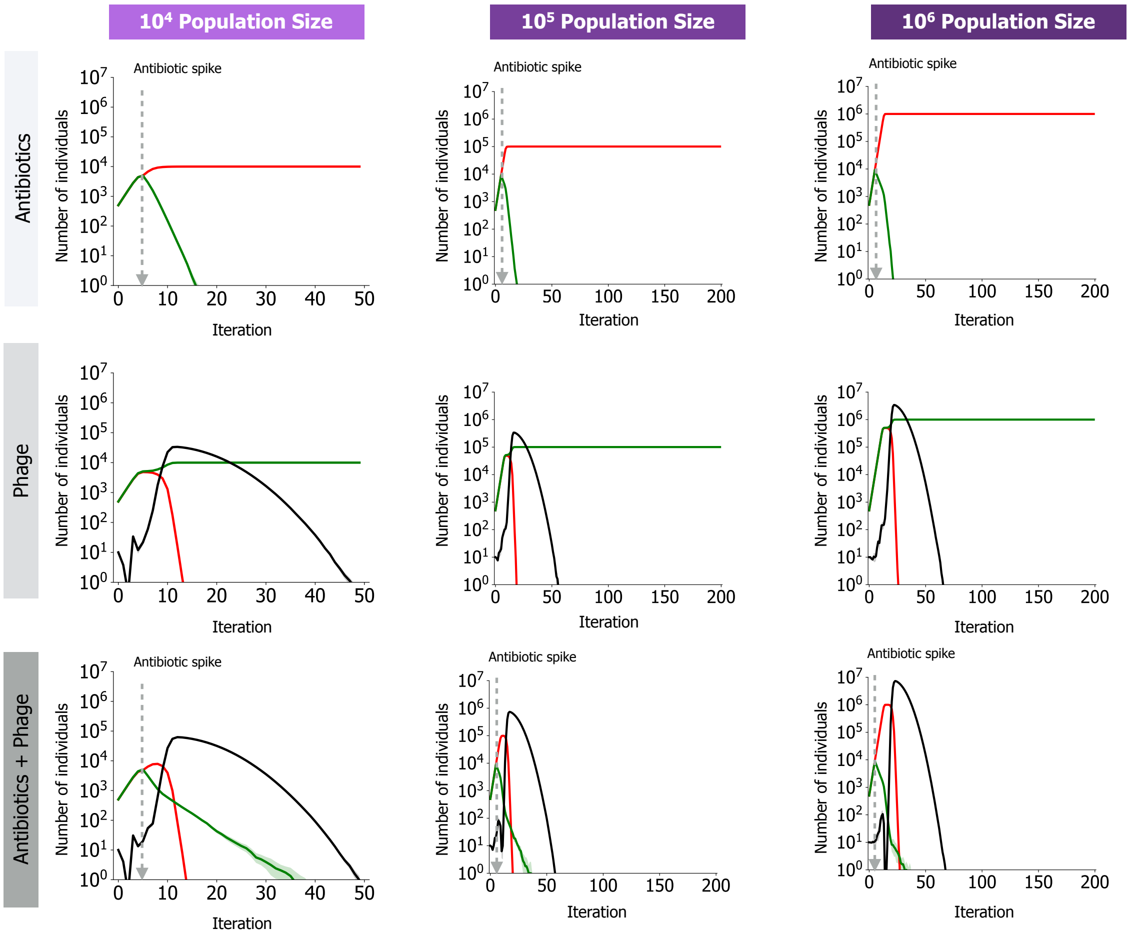

SUPPLEMENTARY FIGURE 18 – The effect of population size in community response to antibiotics, phage, or both, in well-mixed environments. Simulations are similar the ones in Figure 2 of the main text, but with larger population sizes (10^5 and 10^6). 10^4 populations, shown also in Figure 2 (a-c), are displayed here for comparison. Simulations for larger population sizes (10^5 and 10^6) were limited to 6 repeats, due to computational complexity, and have an extended run time of 200 iterations (versus 50 of the 10^4 populations).

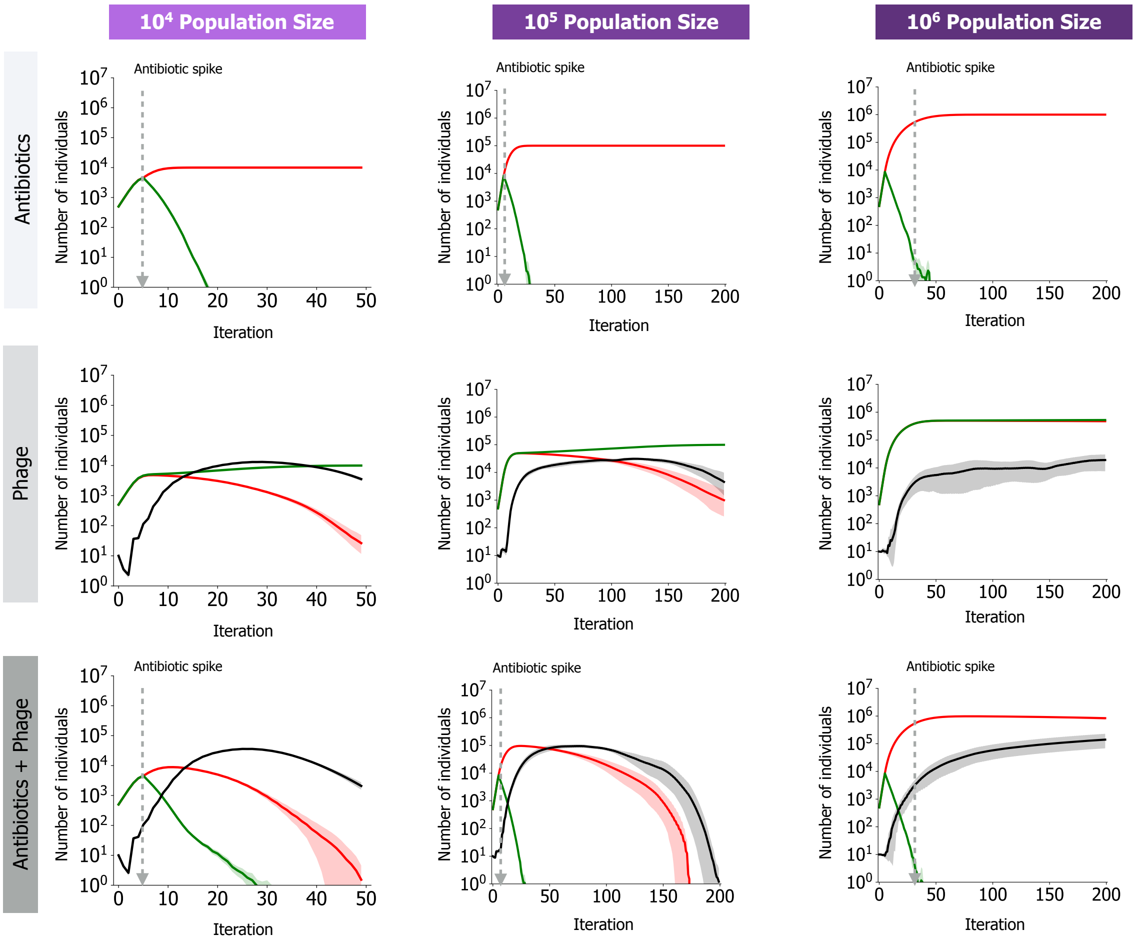

SUPPLEMENTARY FIGURE 19 – **The effect of population size in community response to antibiotics, phage, or both, in spatially structured environments.** Simulations are similar the ones in Figure 2 of the main text, but with larger population sizes (10^5 and 10^6). 10^4 populations, shown also in Figure 2 (d-f), are displayed here for comparison. Simulations for larger population sizes (10^5 and 10^6) were limited to 6 repeats, due to computational complexity, and have an extended run time of 200 iterations (versus 50 of the 10^4 populations). Simulations in structured environments have slower dynamics of extinction due to phage predation (assuming a similar number of initial phages across conditions).

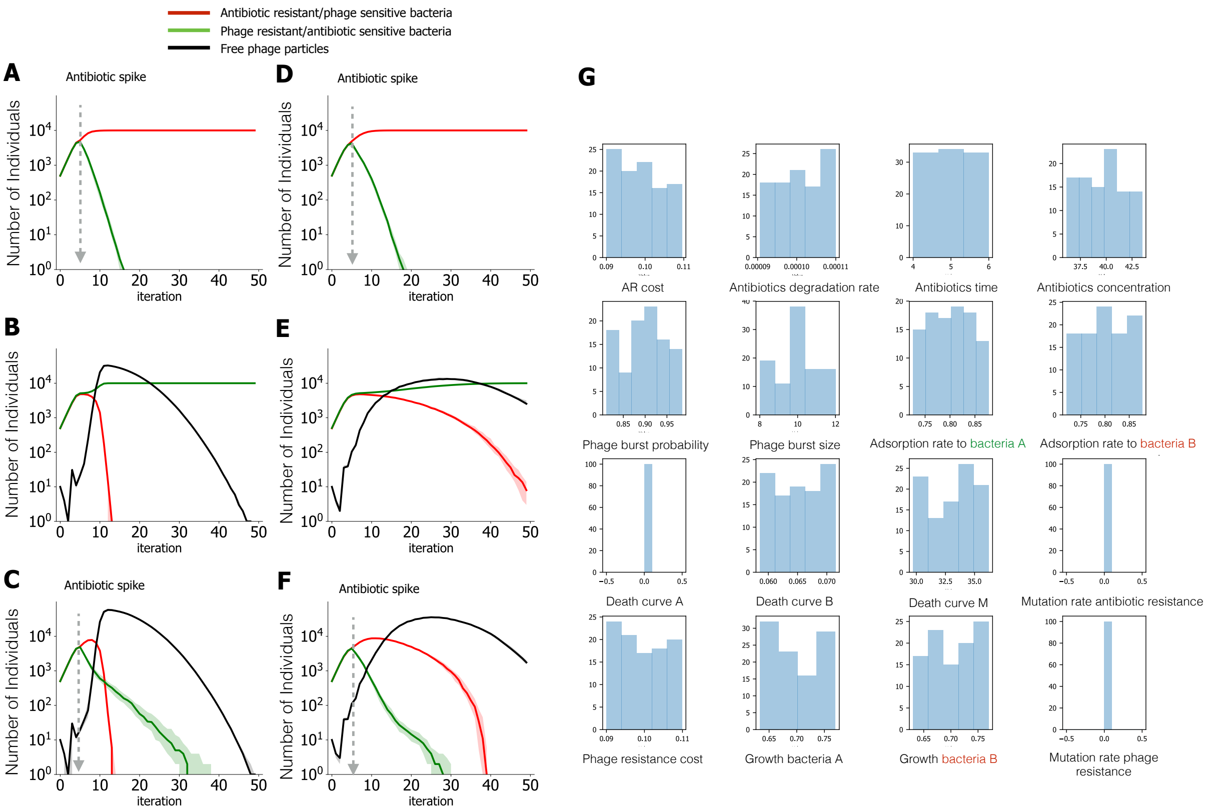

SUPPLEMENTARY FIGURE 20 – **Analysis of robustness for dynamics of phages and antibiotics on a multispecies community.** Conditions of simulations and representation of data in panels (A-F) are similar to the ones simulated for Figure 2 in the main text, but for each of the 100 replicate simulations in each case, parameters are subject to a deviation from the defined value by 10% (deviation value chosen from a uniform distribution). In G are shown the distributions of the parameter used for the 100 replicate simulations for one of the panels (the others have similar distributions).

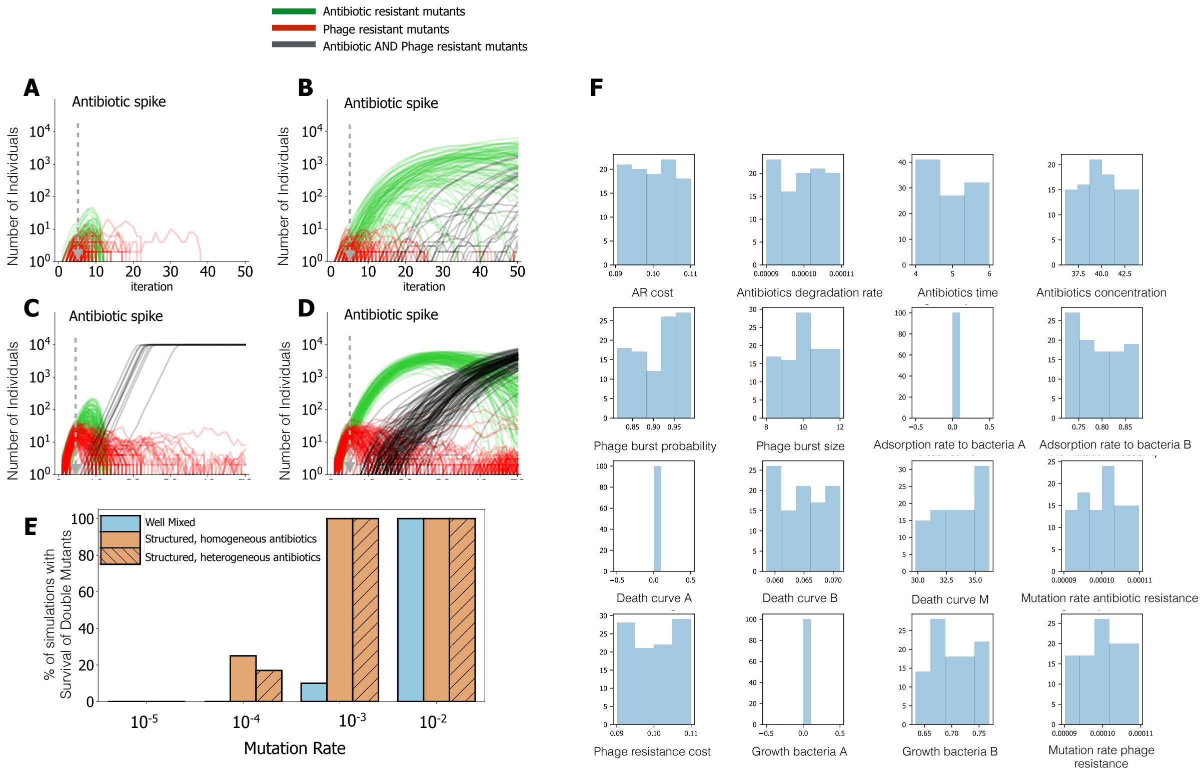

SUPPLEMENTARY FIGURE 21 – Analysis of robustness for the role of structure in the promotion of bacteria resistant to both antibiotics and phages. Conditions of simulations and representation of data in panels (A-E) are similar to the ones simulated for Figure 3 in the main text, but for each of the 100 replicate simulations in each case, parameters are subject to a deviation from the defined value by 10% (deviation value chosen from a uniform distribution). In F are shown the distributions of the parameter used for the 100 replicate simulations in one of the panels. The other have similar distributions, but note that the sub panels "Mutation rate antibiotic resistance" and "Mutation rate phage resistance" are shown for the simulations corresponding to mutation rates of 10^{-4}, and the other cases will have shifted distributions to their respective defined mutation rates.

Supplementary Text S1: The ODD (Overview, Design concepts, and Details) Protocol of the eVIVALDI Model (version 9.2)

1. Overview

1.1 Purpose

The purpose of the model is to explore the role of bacteriophages in the dynamics of microbial communities, given both the role of phage as predatory agents and drivers of horizontal gene transfer between bacteria. The model aims to integrate and study the different mechanisms of predation and transfer (and their interactions) in representing real world scenarios of interactions between bacteria and phage. Additionally, the model's purpose in its current state is also to provide a platform to incrementally add other genomic, ecological and evolutionary mechanisms to study microbial evolution.

1.2 Entities, State Variables and Scales

The model has 7 hierarchical levels: **genes, genomes**, **bacteriophages**, **bacteria, locations, populations** and **environment**. Genomes are comprised of genes and are properties of the two types of agents in the model, bacteria and bacteriophages. Both bacteria and bacteriophage have dynamic genomes, due to the mechanisms of mutation and horizontal gene transfer. Bacteria and bacteriophages exist in a two dimensional environment, where each location (an (x,y) position) can be vacant or occupied by a single bacterium and none to multiple bacteriophage. A free location provides an opportunity for bacteria to reproduce, and represents a form of resource to be competed for.

Below we describe the variables of each hierarchical level, except for the populations, which have no explicit variables.

1.2.1 Genes are identified by their:

- Identifier character (A-Z), which identifies the species to which the gene belongs to in the beginning of the simulation.
- Size (in number of characters, a proxy for base pairs).
- Gene type (Core and/or Mobile).
- Ability to confer antibiotic resistance. If a gene has this ability, an additional phenotype state is included ("Active" or "Inactive").

1.2.2 Genomes are identified by their:

- Ordered list of genes. The initial genome size of bacteria is defined by a global parameter that is common to all species (**Number of genes**).

1.2.3 Bacteriophages are identified by their:

- Phage species name.
- DNA identifier character.
- Lifestyle (temperate, virulent or defective).
- Genome, which excludes the essential phage genes (assumed to be part of the phage). The explicit genome encompasses only a cargo that is composed of either non-essential genes or genes acquired from other bacteria.
- **Maximum cargo size**, the number of genes above which the phage becomes defective, also how many genes can be transmitted by generalized transduction.
- **Attachment morphology**, an internal number that identifies different attachments to bacterial hosts (common to a given species of phages, can be modified, through mutation, by individual phages).
- **Burst size**, identifies how many offspring virions are created from lysing a bacterium.
- **Lysogeny function**, defining the phage decision to integrate into the host genome (common to a given species of phages).
- **Induction function**, defining the phage decision to excise the host genome into which it is integrated (common to a given species of phages).
- **Probability of specialized transduction**, defining the probability of occurrence of this type of transduction during excision (common to a given species of phages).
- **Probability of generalized transduction**, defining the probability of occurrence of this type of transduction during virion packaging (common to a given species of phages).
- Current state (integrated within host, replicating within host, or free living in the environment).
- If integrated in the genome, the genomic position.
- Time, or iterations, passed in the current state (within or outside host).
- An adsorption efficiency towards every bacterial species defined initially (**Probability of adsorption,** specific between a given species of phages and a given species of bacteria).
- A superinfection protection efficiency towards every other phage defined initially (**Superinfection Exclusion Probability,** common to a given species of phages).

1.2.4 Bacteria are identified by their:

- Bacteria species name.
- DNA identifier character.
- Color.
- Genome composition and size (collection of genes and the state of the genes that are mutable).
- Growth rate.
- Location in the 2D grid.
- Resistance to antibiotics (redundant from genomic data).
- List of phage attachment morphologies to which the bacterium is resistant to.
- List of CRISPR cassettes.
- List of phages (currently infecting the bacterium or integrated in its genome as prophages).

1.2.5 Populations are the ensemble of individual bacteriophages and bacteria, and are identified by:

- Subgroups (*e.g.*, populations of the same species, or cells/phages with the same phenotype).
- Number of individuals of each type.

1.2.6 Locations are identified by their:

- 2 dimensional (x,y) position.
- A single bacterium (if the location is occupied by one).
- A number of bacteriophages (if any are located in this particular location).
- The local antibiotic concentration.

1.2.7 The **Environment** is identified by its:

- List of locations.
- Structure (well-mixed, spatially structured or semi-solid). A spatially structured environment fixes the individuals to their locations and the reproduction of bacteria into the Moore neighbourhood (defined as the current location and its 8 adjacent locations in a squared lattice) of distance 1. Abiotic elements (i.e., antibiotics and free phages) do not diffuse from their locations. A semi-solid structure expands the neighbouring Moore distance of reproduction to 3 and creates a diffusion factor of similar distance for the abiotic elements (i.e., antibiotics and free phage) in each location at each time step. In a well-mixed environment, the reproductive distance is kept at Moore distance 1 but the positions of each element in the environment (bacteria, bacteriophage, antibiotics) are randomized immediately after reproduction. (see section 3.3.7 in **Submodels**).
- Continuity (whether the environment is bounded at its borders or toroidal, i.e., the entire environment is a continuum).

1.3 Process Overview and Scheduling

Time is modelled as a succession of discrete iterations at a microbial scale. It is focused on the lifestyle of bacteria, i.e., bacteria cannot replicate more than once per iteration. Iterations much shorter than the minimal doubling time of the fastest growing bacteria in the simulation provide a finer granularity in time (at the expense of computer time).
Bacteria in the simulation are processed in a random order that changes at the beginning of each iteration to avoid biases. If the environment is set as well-mixed, then the location of all bacterial cells is also randomized.

The lifecycle of phage includes calculating the probability of excision (if it is an integrated prophage) and/or burst (if the phage is in the lytic cycle). If excision or burst occurs, it is also at this point that transduction of bacterial genes by phage can occur (see section 3.3.1 in **Submodels**). Upon burst, the offspring of the infecting phage are released into the environment. Once in the environment, phage can immediately infect new bacterial cells, depending on infection efficiency, or it can take more than one iteration for this infection to occur (see section 3.3.5). Importantly, since this process is stochastic, infection is heterogeneous, with some phage infecting faster than others. Infection of temperate phages

is followed by the lysis-lysogeny decision (see section 3.3.6), which defines if the phage integrates the host genome or enters the lytic cycle. Bacteriophage cannot infect and burst a bacterial cell in the same iteration, but prophages can excise and burst within the same iteration.

The death of the bacteria, due to phage predation, antibiotic exposure or stochastic death, leaves their current location vacant, and this resource (space) is free to be consumed by other bacteria (similar to, e.g., [1,2]). Each iteration provides an opportunity for bacteria to reproduce, depending on the availability of these free resources and the growth rate of each bacterium. The speed of reproduction is defined by this growth rate and, due to stochasticity, bacteria with small growth rates can require multiple iterations before producing an offspring, even in the absence of competition. The stochasticity inherent to reproduction leads to an asynchronous reproduction of the bacteria as a population, since some cells can reproduce faster than others. Once a bacterium is selected to reproduce, its offspring will occupy the vacant location. During bacterial reproduction mutations can occur in certain adaptive traits of the offspring (e.g., resistance to antibiotics or to phage), but gene loss can also take place in non-core genes. At each iteration, the process of selection and reproduction occurs for every vacant location, but there is no requirement that every location is filled at each iteration, because a) each bacterial cell can only reproduce once per iteration (if they already reproduced they are unaccounted for in the competition for other locations); and b) no candidates might be suitable to occupy the vacant location, due to either no bacterial cells in the vicinity or very slow growth rate from all the candidate bacteria (see details of this reproductive step in section 3.3.7).

Finally, each iteration also leads to the update of the environment. This includes the degradation of the abiotic elements present in the environment (antibiotics) and free phage, according to the parameterization of the simulations (see section 3.3.8). Additionally, at each iteration cells and abiotic elements are either randomized if the environment is set as well-mixed, or the abiotic elements and phage are diffused locally if the environment is set as semi-solid. The flow chart of the operations at each iteration is represented below.

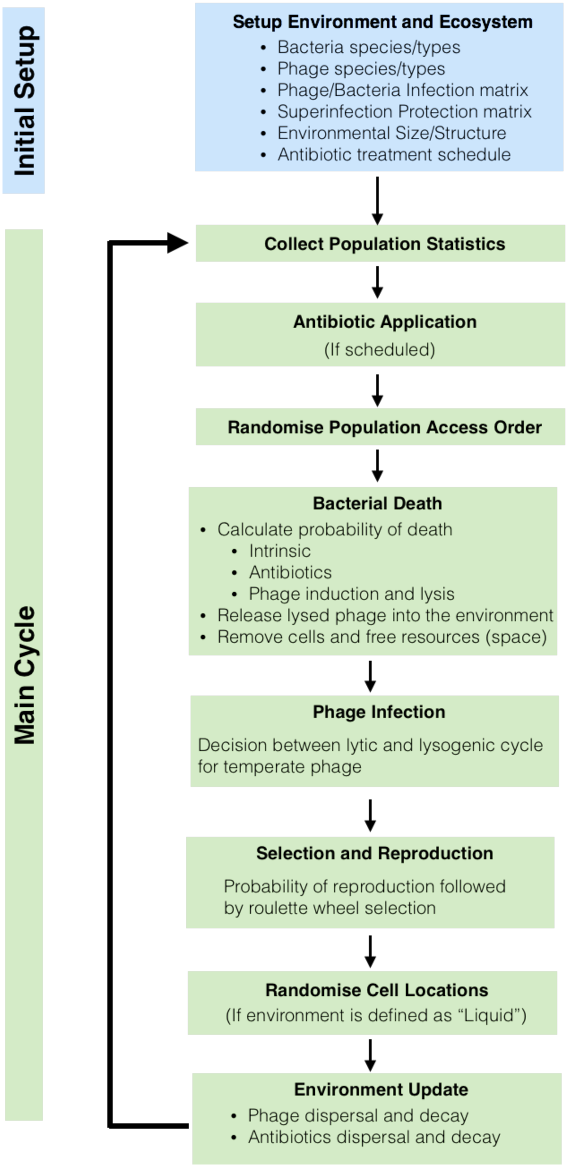

Figure 1 - General flow-chart of the simulations.

2. Design Concepts

2.1 Basic Principles

The model aims to provide qualitative insights on the different mechanisms of interaction between bacteria and bacteriophages, in multi-species communities (of both bacteria and phage) under natural scenarios. The main principle of the model is the dual role of phage in both predating bacteria and serving as a mechanism of horizontal gene transfer. Thus, the main purpose of the model is to study how these two dynamics coexist and in which conditions one will prevail over the other. It is designed with a basis on current knowledge on these interactions, both at the molecular level (e.g., [3]) and at the level of the population dynamics (e.g., [4]). The mechanisms are implemented with the adequate level of detail in order to maintain the model general and computationally feasible.

2.2 Emergence

Population and metapopulation dynamics emerge from the individual behaviour of bacteria and bacteriophage. Phage predation changes the composition of communities based on the phage infection host range (which is defined a priori), but the success of this infection can depend on whether bacteria are within reach of the phage or have become resistant to it. Moreover, in the case of temperate phage, the lysis-lysogeny decision is probabilistically based on the local viral concentration and thus emerges from the individual infections. The outcome of this decision (protection against further infections and use of prophage as weapons) has important, and emergent, effects in the composition of the communities.

The other major emergent aspect of the model is the phage-driven gene flow between bacteria. Phage predation (and bacterial acquisition of resistance to this predation) occurs concurrently with transduction of bacterial genes by phages. Hence, the traits that are transmitted between individual bacteria, the frequency of this transmission, and the way it impacts the survival of the bacteria (i.e., its selective advantage) emerge from the simulations.

2.3 Adaptation

Both bacteria and phage can be subject to mutations in adaptive traits. For bacteria, there are two main selective pressures in the simulations: antibiotics and phage. Genes responsible for antibiotic resistance can switch between the “active” and “inactive” phenotype according to a mutation rate. A different mutation rate can add a particular phage attachment morphology to a list that defines to which phage an individual bacterium can resist infection. For phage, the main selective pressure is on their ability to infect bacterial hosts. During the lytic cycle, genomes of newly formed virions can be different from their progeny because they underwent mutation, at a certain rate, to change their attachment structure morphology, thus being able to escape bacterial defences for infection.

The growth rates of bacteria and the burst size of phage, as well as the diverse rates of mutation, transduction and infection, are immutable, and thus non-evolvable, during the simulations.

2.4 Objectives

The major criteria of success of a bacterial species is its ability to avoid extinction in the face of the selective pressures, antibiotics and phage predation. When in competition with another species, success is also quantified by the frequency of one species over the other(s). A similar criterion can be applied to the phage.

2.5 Learning

There are no learning mechanisms included in this version of the model.

2.6 Sensing

Since the individuals have no access to global variables, such as the population size, both bacteria and phages regulate their behaviour according to the local information (i.e., from current or surrounding locations). Bacteria are affected by the presence of antibiotics in their current location which can modify the probability of death for a bacterium (depending on their phenotypic state regarding antibiotic resistance). Prophages are also indirectly affected by the local antibiotic concentration, as their induction can be mediated the SOS responses activated by, e.g., antibiotic stress [5]. In the simulations, local antibiotic concentrations can increase the probability of prophage induction and excision from sensitive bacteria. The lysis-lysogeny decision is generally believed to favour lysogeny when phages are very abundant, even if there are very few mechanistic studies on the effect of the number of environmental phages on the decision of lysogeny. In phage lambda, lysogeny is favoured under high multiplicity of infection (MOI)[3,6], which is directly proportional to the local phage concentration. In phi3T the probability of lysogeny is a direct function of the density of phages in the local environment by way of an analogue of a quorum-sensing mechanisms [7,8]. In the model, after a successful infection, the lysis-lysogeny decision depends on the viral concentration in the surrounding locations (Moore distance of 1), which is used as a proxy for the MOI (we reasonably assume that if a sensitive bacterium is surrounded by a given number of phage, they will very likely co-infect the cell) in phages that work like phage lambda.

2.7 Interaction

In the model, bacteria-bacteria interactions are always indirect, since no direct contact (from cell A to cell B) is explicit. These indirect interactions occur through competition for resources (space) and through the release of prophage in the environment that kills species B but not species A.

Direct phage-phage interactions occur in the lysis-lysogeny decision, which depends on the local phage concentration [3], and in superinfection exclusion (where prophages exclude infection by similar phages [9,10]).

The infection (and death) of a bacterium cell by a phage is the main direct phage-bacteria interaction. However, the exchange of genes due to transduction, or the injection of transduced DNA by a defective phage are other types of interactions that can occur between these two entities.

2.8 Stochasticity

Microbial communities and environments are highly heterogeneous [11], and the sources of variability are, in the majority of cases, not completely known. This means that some processes are intrinsically stochastic - including mutation-selection-drift - whereas others must be modelled as stochastic because we do not know the sources of variability[12]. The model represents these mechanisms by using stochastic or partly stochastic processes. The majority of these processes are parameterizable, and their frequency can be shaped by changing the respective parameters, studying its consequences for community composition and assessing the similitude with experimental observations.

The parameterizable stochastic processes are:

- Probability of bacterial reproduction (**growth rate** parameter, **resistance costs** parameters and roulette wheel selection process).
- Mutational process (**mutation rate** in the antibiotic resistance gene(s), rate of acquisition of a new morphology to the repertoire of resistances to phage and **probability of gene loss** in bacteria; **mutation rates in receptor or CRISPR sequence in phage**).
- Probability of infection by nearby phage (**adsorption probability** parameter, **infection distance** parameter).
- Probability of bacterial survival to infection by another phage (**superinfection exclusion** parameter).
- Probability of bacterial survival upon infection by a phage caused by CRISPR cassette acquisition (**CRISPR acquisition** parameter).
- Prophage inactivation probability (**inactivation probability** parameter, leads to defective prophage).
- Transduction probability, both specialized and generalized (defined by the parameters **probability of specialized transduction** and **probability of generalized transduction**. The former occurs per phage induction, the latter occurs per virion created during burst).
- Probability of a phage in the lytic pathway to burst (**burst probability** parameter).

There are also processes where the stochasticity of the mechanism is influenced by state variables. These are:

- Lysis-lysogeny decision (2 parameters define a logistic curve as a function of the local viral concentration, **lysogeny alpha** and **lysogeny kappa**, see section 3.3.6).
- Prophage induction decision (2 parameters define a logistic curve as a function of the local antibiotic concentration, **induction alpha** and **induction kappa**, see section 3.3.2).
- Probability of bacterial death and sensitivity to antibiotics (3 parameters define the death function of bacteria, which is a function of the concentration of antibiotics in the bacterium's location, **death curve A**, **death curve B** and **death curve M**, see section 3.3.1).
- Probability of phage elimination (per phage, one parameter defines an exponential decay as a function of phage time outside any bacterial host, **phage decay lambda**, see section 3.3.9).

- Antibiotic decay (per location, one parameter per antibiotic defines an exponential decay as a function of iterations passed since the antibiotic was deployed in a specific location, **antibiotic decay lambda**, see section 3.3.9).

Some other processes are completely random. These are:

- The location of antibiotic resistance gene(s) during genome creation (one gene is selected at random from the entire bacterial genome for each antibiotic).
- The integration site of prophage (a position between genes is chosen at random from the bacterial genome).
- The bacterial DNA transduced during specialized transduction (one gene selected at random from nearby host DNA, where the maximum distance is parameterizable by **Maximum Genomic Distance for Specialized Transduction**).
- The bacterial DNA transferred during generalized transduction (selected at random without replacement from the host DNA, where the number of genes transduced are parameterizable per phage species (**Transduction Max Cargo**), but constant for all generalized transduction events).

Finally, some processes which are presently deterministic could become stochastic in future versions of the model:

- The distance used for reproduction and diffusion in semi-solid environments (fixed at Moore distance 3).
- The number of possible attachment morphologies that phage can express and bacteria can become resistant to (fixed by **possible receptors** parameter).
- The infection distance for phage to infect a bacterial cell (fixed by **infection distance** parameter, set as Moore distance).
- The number of phage offspring after burst (fixed by **burst size** parameter).
- The distance neighbourhood used for the viral concentration assessment in the lysis-lysogeny decision (fixed at Moore distance 1).
- The maximum cargo before a phage becomes defective, defines also how many genes are transduced during generalized transduction (fixed by the **Transduction max cargo** parameter).

In some simulations where the environment is structured instead of well-mixed, the deployment of antibiotics is non-homogeneous. This is a particular mechanism, as it involves operations that are completely random and others with fixed parameters. This is detailed in section 3.3.11 in the **Submodels** section. This (admittedly) complex mechanism was chosen in order to provide an adequate balance between stochasticity and heterogeneity in applying antibiotics to spatially structured environments.

2.9 Collectives

Collective entities are emergent from the model from the groups of individual cells, but no individual decisions take these collectives into account. The collective entities resulting from

the simulations have no state variables or properties, and are meant to evaluate dynamics occurring at the population level resulting from individual mechanisms. They can be assessed at different levels which are, from the highest to lowest level:

- Number of total bacteria or the number of total phage.
- Bacteria from one species or the phage from one species.
- Bacteria with a given phenotype (e.g., antibiotic resistance) or genomic characteristic (e.g., integrated prophage), or phage with a given genomic characteristic (e.g., carrying a transduced antibiotic resistance gene).
- Bacteria in a given cellular state (e.g., ongoing lysis by an infecting phage).

2.10 Observation

The data used for observation of the model is collected mostly from the collective entities described above, regarding the population scale of the simulations. However, depending on the analysis, data can be collected at the level of the individual (e.g., the distribution of the number of phages in bacterial cells) or even at the level of the genomes of individual cells (e.g., the localization of integrated prophages in bacterial genomes). The frequency of data collection can be parameterizable, and it occurs at the beginning of an iteration. The subsequent analysis of these observations can then take into account either all the collected data, or limit the observations to a unique measure to mimic the output of an experimental setting (e.g., the number of bacteria and phage, or the ratio between two bacterial species).

3. Details

3.1 Initialization

Much of the initial state of the simulations can be defined by the experimenter. The random seed itself can also be input as a parameter, in order to achieve reproducible simulations. Initial bacteria and initial phage (if any) are located at random in the environment. The initial properties that can be parameterized are:

- The different bacterial species, and their initial numbers in the population. For each species:
  - Presence/absence of antibiotic resistance phenotypes.
  - Presence/absence of phage resistance phenotypes.
  - Presence/absence of integrated prophage.
- The different phage species, and their initial numbers (in the environment). For each species:
  - Lifestyle (temperate, virulent or defective).
- The type of environment (well-mixed, spatially structured or semi-solid) (**Environmental Structure**).

- The timing and amount of antibiotics applied (per location) (**Antibiotic Application Times** and **Antibiotic Concentration**), as well as the type of antibiotic deployment in spatially structured environments (Homogeneous or heterogeneous, defined in **Antibiotic Arrangement in Structured Environments**).
- The number of possible phage attachment morphologies, defining the phenotypic space of attachment morphology resistance by bacteria (**Number of possible phage receptors**).

Some other properties are fixed depending on the type of simulation required. These include:
- The initial cargo (genes) of phage, and whether it initially includes an antibiotic resistance gene.

3.2 Input Data

The only type of input data used by the model is the time and period of application of antibiotics.

3.3 Submodels

3.3.1 Bacterial Death

Biologically, death in bacterial cells can occur due to antibiotics, either by inhibiting reproduction (bacteriostatic drugs) or by performing lethal biochemical modifications (bactericidal drugs) [13]. Exposure to antibiotics at low concentrations (below the minimum inhibitory concentration) can lead to the death of some cells, but it is compensated by the growth of the population as a whole [14]. Death due to phage occurs by viral replication within the bacterial cell and the subsequent burst of the bacterium. The asymmetrical division of bacterial cells as also been argued to lead to a kind of aging that eventually disrupts the normal function of a bacterium, leading to its death [15]. In the model, we simulate these 3 mechanisms as follows:
- Ageing is simulated by the cumulative probability of intrinsic death (when the antibiotic concentration is zero), which is independent at each iteration but accumulates as time passes. We assume that the individual probability of intrinsic death at each iteration also accounts for other random death events that are not explicitly modelled (e.g., starvation).
- We assume that only bactericidal antibiotics are used in the simulations, and thus contact with the drugs increases the probability of immediate death of a sensitive bacterium. If the bacterium is resistant to antibiotics, the latter have no impact in the probability of death of the former, regardless of their concentration.
- Bacteria infected by phage die when these burst from the cell. Once phage engage with the lytic cycle, the probability of burst (per iteration) is defined by the parameter **Burst Probability**.

The following equation determines the probability of bacterial death at a given iteration, by ageing (in its cumulative form), random death events or antibiotic exposure.

$$DeathProbability = A + \frac{1 - A}{1 + e^{-B*(AntibioticConcentration - M)}}$$

In the absence of antibiotics this results in a non-null minimal probability of death (by design). This function can produce a variety of mortality dynamics, including sigmoid functions as the ones of popular models in pharmacodynamics[16].

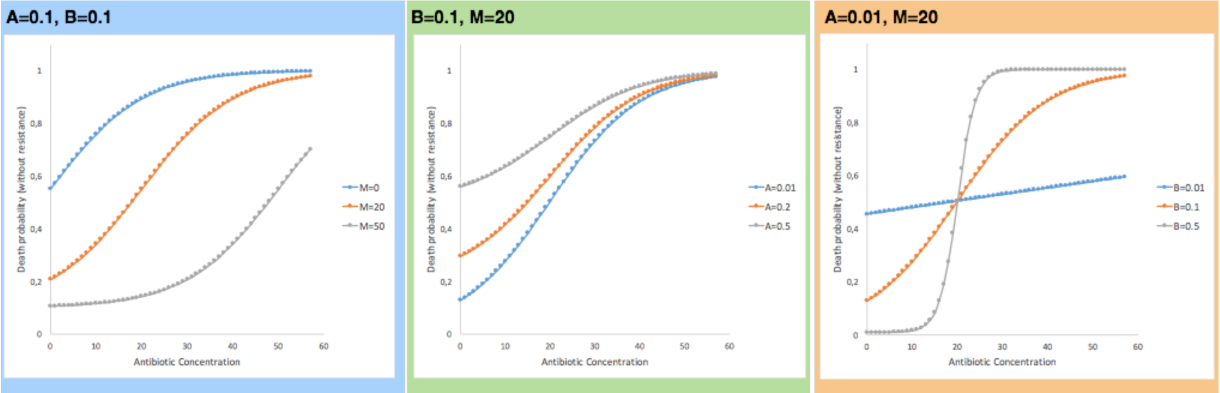

Figure 2 – The different shapes of death curve function, where the probability of death at each iteration (y-axis) is given as a function of the antibiotic concentration (x-axis). When antibiotic concentration is zero, the function corresponds to the intrinsic death probability for each cell.

AntibioticConcentration is the amount of antibiotic present in the current location, and **Death Curve A**, **Death Curve B** **and** **Death Curve M** control the shape of the death function. These 3 parameters allow the experimenter to change the intrinsic death rate (i.e., the probability of death when AntibioticConcentration is zero), the rate of increase in the death probability as the antibiotic concentration increases, and the minimum inhibitory concentration of the drug. Note that by changing any of these parameters, bacteria with different intrinsic mortality rates (when antibiotics are absent) can be studied, allowing the study of this trait in the dynamics of populations[17]. The reasoning for the equation is based on a quasi-logistic function for the sensitivity to antibiotics, where low concentrations lead to a small or null increase in the death rate, then an intermediate region where there is heterogeneity in the death of the individual cells, and finally a region of high antibiotic concentration where bacterial death is certain. However, the three parameters allow the experimenter to explore more flexible functions of response to antibiotics. It can be fitted with experimental data, which shows different concentration-killing curves depending on the antibiotic or the bacteria [18,19]. Finally, if death occurs due to phage burst, the offspring of the infecting phage (the number is defined by the **Burst Size** parameter) will remain in the position where the bacterium was.

3.3.2 Prophage Induction

The induction of a prophage and its entry into the lytic cycle can occur intrinsically, without apparent external stimulus, at low rates [20]. The prophage can also be induced at much higher

rates by external factors, e.g., DNA damage and/or antibiotic exposure [21]. We simulate the induction of prophage according to the following equation, which is a function of the antibiotic concentration:

$$InductionProbability = \frac{1}{1 + \alpha * e^{-AntibioticConcentration+\kappa}}$$

AntibioticConcentration is the antibiotic present in the current location
<u>Induction α</u> and <u>Induction κ</u> control the induction curve

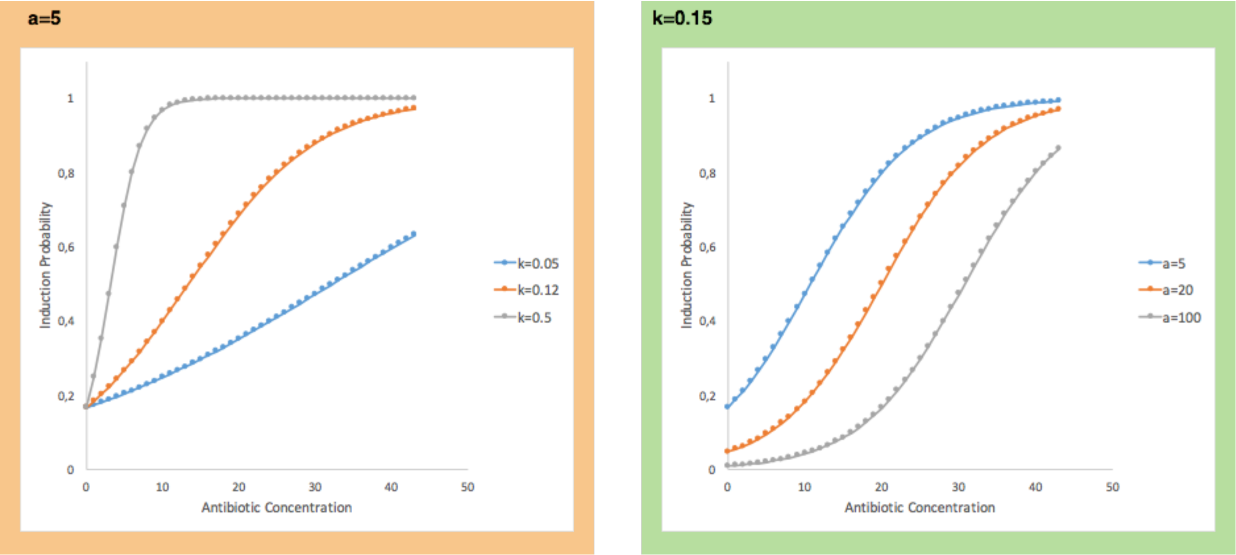

Figure 3 – The different shapes of induction function, where the probability of induction at each iteration (y-axis) is given as a function of the antibiotic concentration (x-axis). When antibiotic concentration is zero, the function corresponds to the intrinsic induction probability for each cell.

The equation represents a logistic equation, as observed experimentally for the induction of phages with ultraviolet or mytomycin C, for instance [22]. The two parameters in the equation control the intrinsic induction probability without antibiotics (parameter **Induction α,** AntibioticConcentration=0) and the necessary antibiotic concentration to induce prophages at a high frequency (mostly defined by **<u>Induction κ</u>**). Induction of prophages can occur under minimum inhibitory concentration of antibiotics, if the induction function defines an increase in probability of induction under a concentration of antibiotics defined as sub-MIC in the death equation in 3.2.1.

3.3.3 Phage Transduction

Transduction by phage can occur by specialized transduction or generalized transduction [23]. The first requires a lysogenic infection, where phage integrate into the bacterial genomes, and thus can occur only with temperate phage. It is characterized by an imprecise prophage excision upon induction of the element, where a piece of adjacent bacterial DNA is excised along with phage DNA. Generalized transduction can occur both with temperate and virulent

phage, since it does not require phage integration. This type of transduction occurs during phage packaging, when a random segment of bacterial DNA, instead of phage DNA, is packaged into the viral capsid. The resulting phage is still able to infect, but since there are no viral genes, it is not able to replicate any longer.

In the model, the probability of specialized transduction is given by the parameter **Specialized Transduction Probability** and is associated with the phage species. Starting from the location of the prophage in the bacterial host genome, one gene will be selected from the list composed of the genes within the **Specialized Transduction Genomic Distance** parameter, in both directions of the genome (upstream and downstream). We assume that non-adjacent genes can be integrated, even though there are adjacent genes that are not. The integrated bacterial DNA can be a core gene, a non-coding gene, a resistance gene, or a piece of another mobile element residing in the genome. The chosen gene is added to the cargo of the phage at the end of its genome. If the total number of genes in the phage cargo after transduction (considering both the existing genes and the transduced ones) is above **Transduction Max Cargo Size** parameter, then the phage becomes defective. Since this occurs during excision, and before replication of the phage, all the virion offspring will carry the same transduced DNA.

Generalised Transduction is not necessarily associated with lysogeny (it can be undertaken by both temperate and virulent phage). Contrary to specialized transduction, where all virions will have the same transduced bacterial DNA, each virion produced might undergo generalised transduction independently, with **Generalised Transduction Probability**, which is defined for each phage species. Thus, if more than one generalized transduction event occurs during a single burst, the transducing virions are likely to carry different segments of bacterial DNA. The number of bacterial genes transduced is assumed to be always the same, but is specific for each phage species, and is defined by the **Transduction Max Cargo Size** parameter. This fits experimental data showing that the amount of packaged DNA varies on a narrow range and is a function of capsid size [24]. The actual bacterial genes transduced are chosen randomly from the entire genome (and may include other mobile elements, including other genes contained in other phage cargo). The current cargo of the virion is completely replaced by this set of genes and the phage becomes defective.

Defective phages that are released into the environment (after either specialized or generalized transduction) are assumed to still be able to infect bacterial cells with the same efficiency of its non-defective variant, and integrate the transduced DNA into the bacterial genome. However, they cannot be induced, excise, or replicate within the bacteria.

3.3.4 Phage Mutation

Phages are known to have higher mutation rates than their hosts [25], and although this leads to a high load of deleterious mutations, it is also a key process in their ability to keep up with the evolution of bacterial defences to phage infection [4]. Mutations in phage are known to allow them to escape bacterial defences based on changes on their tail fibers responsible for attaching to bacteria [26] or mutations in regions previously acquired by the bacteria as CRISPR cassettes [27].

In our simulations, we assume that beneficial, neutral or detrimental mutations can occur in phages, related with the ability of phage to escape bacterial defences and that and they take place during viral replication. First, virions can acquire mutations that change the morphology of their bacterial receptor. This does not represent a change in the host range of the phage, but instead allows them to successfully attach to their hosts (beneficial mutations). This occurs with a probability given by the parameter **Phage Receptor Mutation Probability**. The receptor morphology is characterized by a number, and the maximum number of receptors possible (i.e., the possible receptor phenotypes) is given the parameter **Possible Receptors**. Upon mutation, a random sample is performed of the values comprised between 0 and **Possible Receptors** (with the exception of the current receptor), and this new receptor morphology is assigned to the mutated phage.

From the point of view of the bacteria, we assume that CRISPR-based defences (see section 3.3.5 below) is based on the acquisition of a particular sequence present in each individual phage (a 10-character ASCII string). Virions can thus mutate this sequence, allowing them to infect bacteria that have previously acquired their (original) CRISPR sequence. This occurs with a probability given by the parameter **Phage CRISPR Mutation Probability**, and results in the random generation of a new 10-character string.

3.3.5 Phage Infection

The process of infection of a bacterium by a phage involves multiple processes, spanning the physical proximity between the two, the ability of phage to attach to the bacterial cell, and the injection of viral DNA into the bacterial host. Multiple mechanisms can prevent phage infection, including (but not limited to) avoiding contact with the phage [28], acquiring mutations that change the surface of the receptor responsible for phage attachment [29], or the presence of other phage in the cell prevents the injection and expression of invading viral DNA [9,10]. Accordingly, the model attempts to reproduce infection as a multi-step process to allow the introduction of these mechanisms. The infection of a bacterial cell by a phage is first constrained by the proximity between the two entities. The minimum distance for infection is given by the parameter **Infection Distance**, which is measured as a Moore distance necessary for the contact between phage and bacteria. An **Infection Distance** of zero means that phage can only infect bacteria that are located in the same position as the phage. After this initial contact, a series of barriers have to be overcome:

- The probability of phage adsorption to the cell is given by the **Adsorption Probability** of the phage species in the bacterial cell's species (i.e., the host range of the phage). Hence, some bacteria-phage interactions are much more likely to result in successful attachment at each iteration than others.
- Following attachment, the phage must surpass bacterial defences. They are, in the order they are checked:
    - Superinfection probability, represents the probability that a phage succeeds in infecting a bacterium that already harbors a phage. It is defined at the beginning of the simulations in the form of a matrix characterizing all pairwise interactions between the different types of phage. If more than

one resident phage can provide superinfection protection, the success of an infection is assumed to be given by the one that can provide the highest amount of protection against the infecting phage.
    - Receptor defences, where bacteria must not have acquired mutations that prevent the phage infection (see section 3.3.9).
    - CRISPR defences, where bacteria may have acquired a CRISPR cassette that prevents phage infection. The acquisition of CRISPR cassettes can occur during an infection (if so, the infection fails and the phage is removed from the environment), and the bacterium adds the 10 character ASCII string representing the phage sequence to its CRISPR array.
- If infection failed due to receptor defenses or superinfection exclusion, the phage is kept in the environment, where it might attempt another infection to other bacteria (in the same iteration) or the same one (in the next iterations). On the other hand, bacterial CRISPR acquisition of an invading phage always removes the phage from the environment.
- If the bacterium is unable to use any of the defence mechanisms to prevent phage infection, then the phage is removed from the environment and it becomes part of the bacterial entity.

Once the infection has been successful, the counter of the number of iterations the phage has spent outside a host is reset to zero. The phage lifestyle defines what occurs next. Virulent phage will necessarily enter the lytic cycle, start replicating and prepare to burst (we assume that burst cannot occur in the same iteration as the infection). A temperate phage performs a lysis-lysogeny decision (see below). If the lytic pathway is chosen, then it becomes “Induced temperate” and it enters the lytic cycle, starts replicating and preparing to burst, like virulent phages do. If the lysogenic pathway is chosen, or if the incoming phage is defective, then the DNA carried by the viral particle integrates into the bacterial host genome, at a randomly chosen position.

3.3.6 Phage Lysis-Lysogeny Decision

The determinants of the lysis-lysogeny decision are not completely known, and are the subject of intensive research. The decision seems to involve some information on the amount of potentially competing phages, *e.g.*, through MOI or quorum-sensed based information on the number of phages in the environment [6 8]. High values of these variables increase the probability of lysogeny (but the process remains stochastic). Exactly how this assessment is performed is most often not clear. Thus, this decision is simulated by taking into account the local viral concentration (i.e., number of phage within Moore distance 1) which is a proxy for the presumptive MOI in the host bacteria. It follows a probabilistic logistic equation, as observed experimentally [3]. The equation chosen to model this decision allows to control the intrinsic rate of lysogeny (i.e., when there are no phage), and how fast the probability increases with an increasing number of surrounding phage. The equation is as follows:

$$LysogenyProbability = \frac{1}{1 + \alpha * e^{-SurroundingPhage + \kappa}}$$

SurroundingPhage is the number of phage within Moore distance 1
α **and** κ control the lysogeny curve

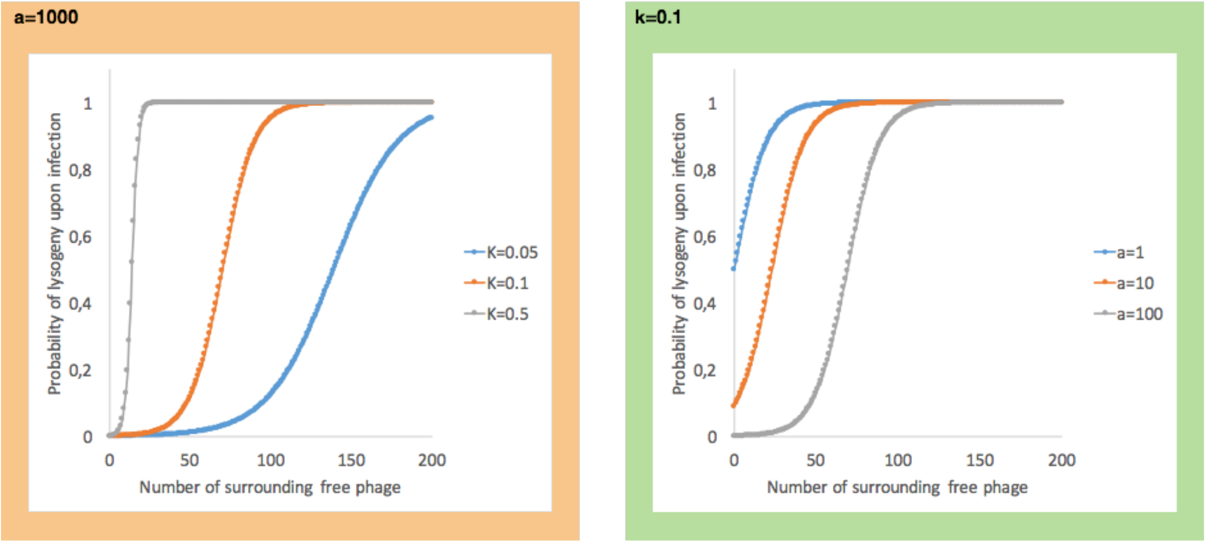

Figure 4 – The different shapes of lysogeny function, where the probability of lysogeny at each iteration (y-axis) is given as a function of the number of phage in the Moore distance 1 (x-axis). When the number of phage is zero, the function corresponds to the intrinsic lysogeny probability for each phage.

3.3.7 Bacterial Reproduction

At each iteration, and for every vacant location, a competition between eligible bacteria defines which one (if any) will produce an offspring to occupy the location. There are four criteria for eligibility.

1) **Distance**. Bacteria must be less than a maximum distance from the vacant location (Moore distance of 1 for spatially structured or well-mixed and of 3 for semi-solid structure). The decision of keeping a distance of 1 for well-mixed environments rests on the assumption that cells do reproduce locally even in liquid environments, and it is the absence of structure that will quickly, and in a potential far-reaching distance, separate mother and daughter cells (which we reproduce by randomizing all cell positions at each iteration immediately after the reproductive cycle of the entire population). The Moore distance 3 for a semi-solid environment, on the other hand, represents a (potential) limited diffusion between the cells after reproduction.

2) **Growth rate**. The growth rate defined by the experimenter is corrected to account for the cost of resistances to antibiotics or phages (if relevant), resulting in a compound growth rate representing the fitness of the individual cell. These costs are also parameterizable by the experimenter, and affect the original growth rate in a multiplicative way:

$$GrowthRate_{Compound} = \begin{cases} GrowthRate * (1 - CostPhageResistance), & \text{if resistant to phage (receptor)} \\ GrowthRate * (1 - CostAntibioticResistance), & \text{if resistant to antibiotics (chromosomal)} \\ GrowthRate * (1 - CostAntibioticResistance) * (1 - CostPhageResistance), & \text{if resistant to antibiotics and phage} \\ GrowthRate, & \text{otherwise} \end{cases}$$

Based on this compound growth rate, cells can be discarded from entering the competition for reproduction in the current iteration. This decision is stochastic, meaning that cells with a low compound growth rate will enter the competitive round of reproduction less often. If the population is homogeneous (i.e., the growth rate is similar), this results in a slower growth of the population, since more iterations are required for the entire population to double in size. This decision depends solely in the growth rate of cells (and eventual costs), and has no account for other competitor bacteria.

3) **Previous reproduction**. The bacterium cannot have previously reproduced in the current iteration.

4) **Lysis**. The bacterium must not be infected by a phage that is producing lysis (virulent or induced temperate phage). If this is the case, the bacterium is ineligible to reproduce.

If, after these four conditions, no bacteria are eligible to reproduce and occupy the vacant space, this space remains vacant at least until the next iteration. If there are eligible bacteria, all of them will enter into direct competition for reproduction. Their compound growth rate is used in a roulette wheel process to choose the winning candidate. In the roulette wheel selection process, the competitive fitness (compound growth rate) of bacterium *i* is proportional to its probability of being selected to reproduce to the empty location, hence their probability of reproduction (higher for fitter cells) is influenced by their competitors. At every iteration, this process is repeated until all vacant locations are processed, but not necessarily occupied.

The model fundamental unit of time is the iteration. The classical concept of bacterial generation, as considered in population genetics, is not explicitly modelled here because it is not individual-centred, no evidence of frequent synchronization of replication in nature, and because there may be many different species with different growth rates in the simulations. Instead, the biological meaning of "generation" for a given species emerges from the simulations. If one wishes to translate the notion of iteration to that of generation one should consider that a growth rate of (e.g.) 0.5 (meaning 50% probability of replication in the current generation) implies that a cell with access to resources (space) and in the absence of competition divides, on average, every two time-steps (iterations). However, a generation in terms of the population is a compound factor of the effective growth rate and of the mortality rate of the individual cells that are the result of the amount of resources available, the presence of stressors (that increase mortality) and the population size (competition for resources). The ensemble of these computations across all individual cells will result in a population growth rate, and this (along with the time needed for a doubling of the number of bacteria) will change as the populations change in size and the availability of resources (space) changes. Without any stressors (i.e., antibiotics or phage predation), bacteria will divide until they reach the limit of resources (i.e., the number of available individual spaces. However, at this stationary demographic state, fluctuations can occur in the model due to death of existing cells, liberation of their resources (space), and their replacement by new

births that occur when resources are made available. In the example shown in Figure 5 (green line) below, 100 bacteria, of a species whose growth rate is 0.5, were inoculated at time 0. According to the classical calculation of the number of generations ($2^n \times B_0 = B_{final}$, solving for n), it took ~13.3 generations to reach stable demography (until iteration 18), or 0.74 generations per iteration. For a slightly lower growth rate (red bacteria) 13.1 generations elapsed in the same number of iterations, whilst a higher mortality rate (blue line) show a lower number of generations (~10) even if the growth rate is similar to the ones in the bacteria from the green line. This shows that different cellular parameters (division and mortality) account not only for the fate of an individual cell, but also for the assessment of population-level dynamics such as the generation time.

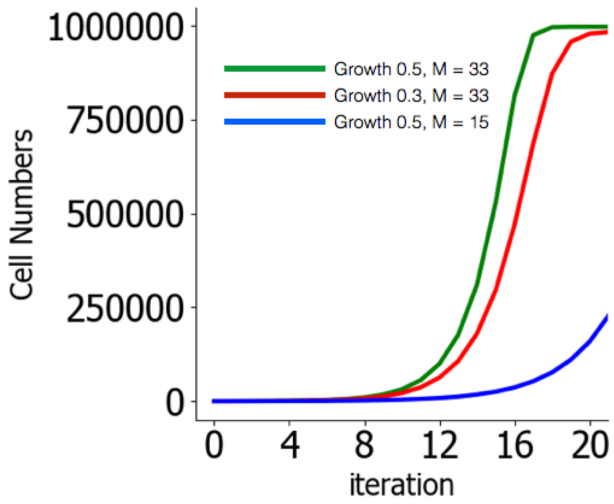

Figure 5 – Typical growth curves obtained with the model, for bacteria with different parameters growing in well-mixed environments. At time 0, 100 cells of bacteria were inoculated and grown for 22 iterations. For these simulations the limiting resource, or the number of individual locations in the environment, is 10^6. Green and blue lines show bacteria with similar growth rates but a higher mortality rate affects the bacteria represented by the blue lines. Red line shows bacteria with a mortality rate similar to green bacteria, but with a lower growth rate.

The previous observation is also associated with the concept of "iteration" (the atomic time-step of the model), whose biological meaning might not be immediately obvious. In essence, the experimenter can define the time equivalent of an iteration by changing the growth parameter of a bacterial species. For instance, if one considers *Escherichia coli*, which has been observed to divide (under optimal growth conditions) every 20 minutes, and if a growth rate of 0.5 corresponds to a division every (average) 2 iterations, then one iteration will correspond roughly to 10 minutes.

3.3.8 Bacterial Mutation

When reproducing, bacteria generate offspring that are identical copies of themselves, including the genome, the (eventual) phage, and CRISPR cassettes. However, some traits can undergo a mutational process to differ from the parent cell. These are:

- Antibiotic resistance genes, which can switch between the "inactive" and "active" state according to the (same) probability given by the parameter **ARG Mutation Probability**. This is assessed in a gene-by-gene fashion, where all putative resistance genes, be it on the original genome or on genes carried by phage, may mutate.
- List of phage receptors morphologies avoided by the bacterium, which can increase according to the probability given by the parameter **Resistance Phage Receptor Mutation Probability**. As for the mutations in the phage themselves, the maximum number of receptors possible (i.e., the possible receptor phenotypes) is given the parameter **Possible Receptors**. Upon mutation, a random sample is performed from the values comprised between 0 and **Possible Receptors** (with the exception of the receptors that are already in the list of the bacterium), and this new receptor is added to the list of resistances.
- Gene loss. Non-core or mobile elements can be deleted from the genome with probability (per gene) given by the parameter **Gene Loss Probability**.

3.3.9 Environmental Degradation

In the model, there are two elements subject to degradation through time: antibiotics and phage particles outside bacterial hosts.

Antibiotics are degraded with an exponential decay, assuming first-order kinetics following observations from the literature[30], with parameter **Antibiotic Degradation Lambda** (independent for each type of antibiotic), and their concentration decreases along time following the equation:

$$Concentration_{t+1} = Concentration_0 * e^{-\lambda * Concentration_t}$$

Concentration is the antibiotics concentration in the current location
t is the current iteration
λ is the decay factor

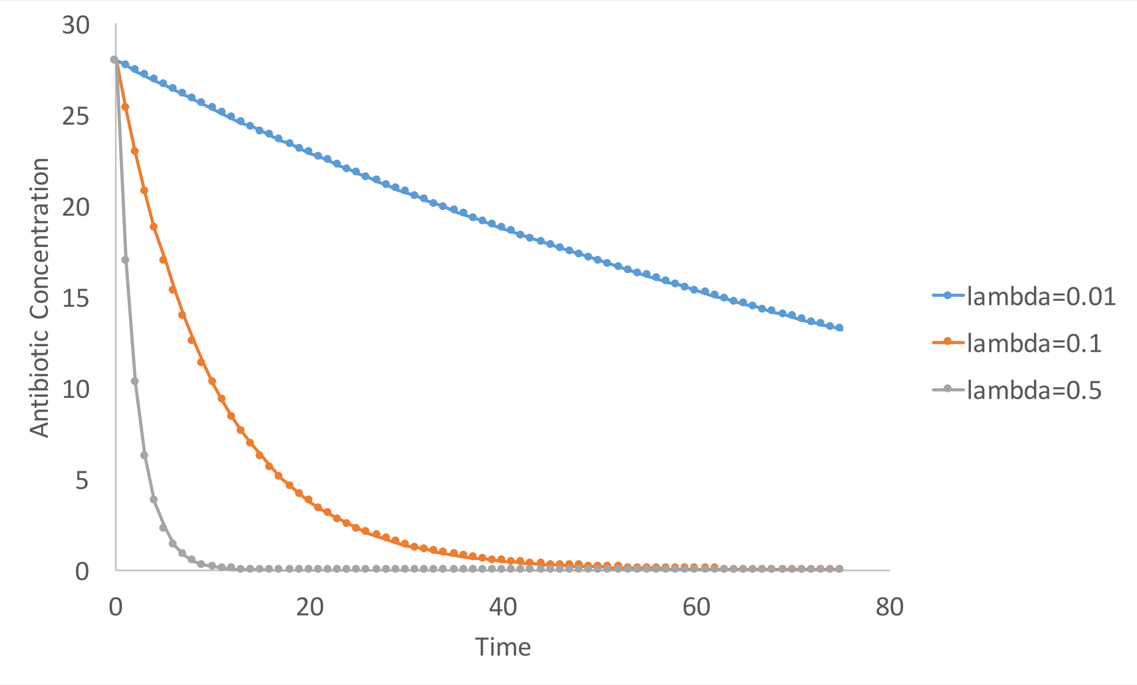

Figure 6 – The antibiotic decay function, where the antibiotic concentration (y-axis) is decreased as function of time (x-axis). Different rates of decay are obtained with different lambda parameters.

Individual phage particles disappear along time also according to an exponential-decay function[31]. However, since there is no continuous degradation of the phage, this function gives the probability for a single phage to disappear from the environment according to the time that it has lived in the environment (i.e., outside a host). In terms of population size, this implies also a quasi-exponential decay (due to stochasticity). The probability of phage elimination from the environment was approximated by the equation:

$$PhageSurvivalProbability = 1 * e^{-\lambda * TimeOutsideHost}$$

TimeOutsideHost is the time the phage has spent in the environment
λ is the decay factor

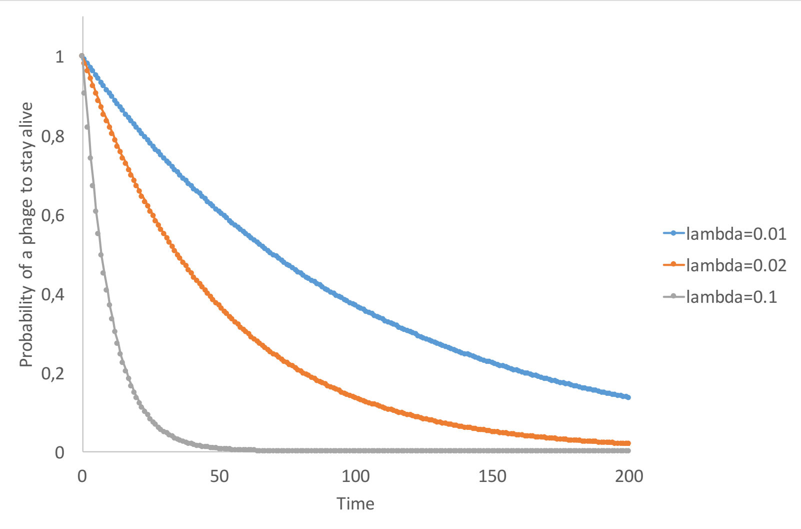

Figure 7 – The phages' elimination (and phage population decay) function, where a single phage's probability of being kept in the environment decreases as function of time it has spent outside a host (x-axis). Different rates of population decay are obtained with different lambda parameters.

3.3.10 Environmental Diffusion

Diffusion of phage particles and antibiotics occurs when the environment is set as well-mixed or semi-solid.

For well-mixed environments, the antibiotic concentrations of each location are randomly re-assorted to new locations, since we assume a homogeneous diffusion of the drug. Free phages are randomly re-assorted to new positions if there are fewer phages than the total number of locations. Otherwise, a uniform re-distribution, or as uniform as possible (i.e., if after a uniform distribution of the majority of the phages, the remaining ones, if any, are allocated randomly to a location), of the phage for all the locations is performed. This allows a homogeneous distribution of the phage throughout the environment.

For semi-solid environments, both antibiotics and phage are locally, but randomly, switched amongst locations within Moore distance 3.

In environments that are spatially structured, phages can only diffuse between cells upon infecting a bacterial cell, whilst antibiotics do not diffuse.

3.3.11 Antibiotic placement in spatially structured environments

The arrangement of antibiotics in spatially structured environments is defined by the parameter **Antibiotic Arrangement in Structured Environments**. If this is set as "Homogeneous", antibiotics are deployed as in well-mixed environments (each location harbours the same antibiotic concentration). On the other hand, "Heterogeneous" antibiotic arrangements create stochastic landscapes of antibiotic concentrations across the environment in the following way:

- First, a random sample (with replacement) of half of all locations is performed.
- Second, an antibiotic concentration (with the value randomly chosen between the maximum antibiotic concentration defined and half of this value) is deposited in each chosen location.
- Third, diffusion occurs recursively for each location with antibiotics, with the diffused antibiotic concentration that reaches a new location being randomly chosen between X and X/2 (with X=exponential decayed (with lambda 0.17) antibiotic concentration from the location where the diffusion originates).
- Finally, the recursive diffusion occurs while the antibiotic concentration in the new location is above 1.

REFERENCES

1. Werisch, M., Berger, U. & Berendonk, T. U. Conjugative plasmids enable the maintenance of low cost non-transmissible plasmids. *Plasmid* **91,** 96–104 (2017).
2. Haerter, J. O., Trusina, A. & Sneppen, K. Targeted bacterial immunity buffers phage diversity. *J. Virol.* **85,** 10554–10560 (2011).
3. Zeng, L. *et al.* Decision making at a subcellular level determines the outcome of bacteriophage infection. *Cell* **141,** 682–691 (2010).
4. Weitz, J. S., Hartman, H. & Levin, S. A. Coevolutionary arms races between bacteria and bacteriophage. *Proc. Natl. Acad. Sci. U.S.A.* **102,** 9535–9540 (2005).
5. Lamont, I., Brumby, A. M. & Egan, J. B. UV induction of coliphage 186: prophage induction as an SOS function. *Proc. Natl. Acad. Sci. U.S.A.* **86,** 5492–5496 (1989).
6. Kourilsky, P. Lysogenization by bacteriophage lambda. I. Multiple infection and the lysogenic response. *Mol. Gen. Genet.* **122,** 183–195 (1973).
7. St-Pierre, F. & Endy, D. Determination of cell fate selection during phage lambda infection. *Proc. Natl. Acad. Sci. U.S.A.* **105,** 20705–20710 (2008).
8. Erez, Z. *et al.* Communication between viruses guides lysis-lysogeny decisions. *Nature* **541,** 488–493 (2017).
9. Susskind, M. M., Botstein, D. & Wright, A. Superinfection exclusion by P22 prophage in lysogens of Salmonella typhimurium. III. Failure of superinfecting phage DNA to enter sieA+ lysogens. *Virology* **62,** 350–366 (1974).
10. Bondy-Denomy, J. *et al.* Prophages mediate defense against phage infection through diverse mechanisms. *ISME J* **10,** 2854–2866 (2016).
11. Ackermann, M. A functional perspective on phenotypic heterogeneity in microorganisms. *Nat Rev Micro* **13,** 497–508 (2015).
12. Golding, I. Infection by bacteriophage lambda: an evolving paradigm for cellular individuality. *Curr Opin Microbiol* **43,** 9–13 (2018).
13. Kohanski, M. A., Dwyer, D. J. & Collins, J. J. How antibiotics kill bacteria: from targets to networks. *Nat Rev Micro* **8,** 423–435 (2010).
14. Davies, J., Spiegelman, G. B. & Yim, G. The world of subinhibitory antibiotic concentrations. *Curr Opin Microbiol* **9,** 445–453 (2006).
15. Ackermann, M., Stearns, S. C. & Jenal, U. Senescence in a bacterium with asymmetric division. *Science* **300,** 1920–1920 (2003).
16. Demidenko, E., Glaholt, S. P., Kyker-Snowman, E., Shaw, J. R. & Chen, C. Y. Single toxin dose-response models revisited. *Toxicol. Appl. Pharmacol.* **314,** 12–23 (2017).
17. Servais, P., Billen, G. & Rego, J. V. Rate of bacterial mortality in aquatic environments. *Appl. Environ. Microbiol.* **49,** 1448–1454 (1985).
18. Liu, Y. Q., Zhang, Y. Z. & Gao, P. J. Novel concentration-killing curve method for estimation of bactericidal potency of antibiotics in an in vitro dynamic model. *Antimicrob Agents Chemother* **48,** 3884–3891 (2004).
19. Levin, B. R. & Udekwu, K. I. Population dynamics of antibiotic treatment: a mathematical model and hypotheses for time-kill and continuous-culture

experiments. *Antimicrob Agents Chemother* **54,** 3414–3426 (2010).
20. Nanda, A. M., Thormann, K. & Frunzke, J. Impact of spontaneous prophage induction on the fitness of bacterial populations and host-microbe interactions. *J Bacteriol* **197,** 410–419 (2015).
21. Oppenheim, A. B., Kobiler, O., Stavans, J., Court, D. L. & Adhya, S. Switches in bacteriophage lambda development. *Annu. Rev. Genet.* **39,** 409–429 (2005).
22. Castellazzi, M., George, J. & Buttin, G. Prophage induction and cell division in E. coli. II. Linked (recA, zab) and unlinked (lex) suppressors of tif-1-mediated induction and filamentation. *Mol. Gen. Genet.* **119,** 153–174 (1972).
23. Touchon, M., Moura de Sousa, J. A. & Rocha, E. P. Embracing the enemy: the diversification of microbial gene repertoires by phage-mediated horizontal gene transfer. *Curr Opin Microbiol* **38,** 66–73 (2017).
24. Ikeda, H. & Tomizawa, J. I. Transducing fragments in generalized transduction by phage P1. 3. Studies with small phage particles. *J. Mol. Biol.* **14,** 120–129 (1965).
25. Drake, J. W. Comparative rates of spontaneous mutation. *Nature* **221,** 1132 (1969).
26. Meyer, J. R. *et al.* Repeatability and contingency in the evolution of a key innovation in phage lambda. *Science* **335,** 428–432 (2012).
27. Semenova, E. *et al.* Interference by clustered regularly interspaced short palindromic repeat (CRISPR) RNA is governed by a seed sequence. *Proc. Natl. Acad. Sci. U.S.A.* **108,** 10098–10103 (2011).
28. Simmons, M., Drescher, K., Nadell, C. D. & Bucci, V. Phage mobility is a core determinant of phage-bacteria coexistence in biofilms. *ISME J* **89,** 135 (2017).
29. Labrie, S. J., Samson, J. E. & Moineau, S. Bacteriophage resistance mechanisms. *Nat Rev Micro* **8,** 317–327 (2010).
30. Pan, M. & Chu, L. M. Adsorption and degradation of five selected antibiotics in agricultural soil. *Sci. Total Environ.* **545-546,** 48–56 (2016).
31. De Paepe, M. & Taddei, F. Viruses' life history: towards a mechanistic basis of a trade-off between survival and reproduction among phages. *PLoS Biol.* **4,** e193 (2006).
